# Ultrafast Water H-Bond Rearrangement in a Metal–Organic Framework Probed by Femtosecond Time-Resolved Infrared Spectroscopy

**DOI:** 10.1021/jacs.3c01728

**Published:** 2023-05-18

**Authors:** Mason L. Valentine, Guoxin Yin, Julius J. Oppenheim, Mircea Dincǎ, Wei Xiong

**Affiliations:** † Department of Chemistry and Biochemistry, University of California San Diego, La Jolla, California 92093, United States; ‡ Materials Science and Engineering Program, University of California San Diego, La Jolla, California 92093, United States; § Department of Chemistry, Massachusetts Institute of Technology, 77 Massachusetts Avenue, Cambridge, Massachusetts 02139, United States

## Abstract

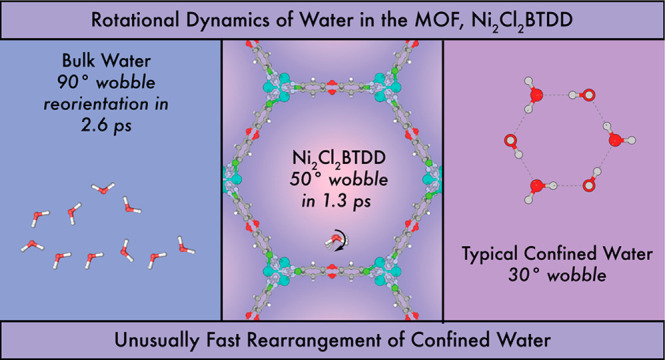

We investigated the water H-bond network and its dynamics in Ni_2_Cl_2_BTDD, a prototypical MOF for atmospheric water harvesting, using linear and ultrafast IR spectroscopy. Utilizing isotopic labeling and infrared spectroscopy, we found that water forms an extensive H-bonding network in Ni_2_Cl_2_BTDD. Further investigation with ultrafast spectroscopy revealed that water can reorient in a confined cone up to ∼50° within 1.3 ps. This large angle reorientation indicates H-bond rearrangement, similar to bulk water. Thus, although the water H-bond network is confined in Ni_2_Cl_2_BTDD, different from other confined systems, H-bond rearrangement is not hindered. The picosecond H-bond rearrangement in Ni_2_Cl_2_BTDD corroborates its reversibility with minimal hysteresis in water sorption.

Freshwater scarcity is a growing problem due to pollution, increased urban density, and the exhaustion of freshwater sources. Materials based on metal–organic frameworks (MOFs) have been pursued in water processing and recycling. MOFs are highly porous tunable materials formed through the self-assembly of organic linkers and metal clusters. The resulting well-defined pores, up to several nanometers in diameter, give MOFs the highest surface areas measured to date, making them appealing for water processing.^1^ Many MOF-based acquisitions of fresh water^2−7^ rely on specific control of MOF–water interactions. Particularly, atmospheric water harvesting (AWH)^8^ requires fast and reversible water sorption over a narrow and convenient humidity range (10–30%), which demands exquisite manipulation of water–water and water–framework interactions.^9,10^

Although fundamental physical studies of water in MOF pores have led to the development of more efficient MOFs for AWH,^11−14^ there are significant barriers to understanding water H-bond networks and dynamics in MOFs, as water molecules may not behave the same way when confined over nanometer length scales as they do in bulk.^15−20^ These challenges are rooted in the characterization methods for MOFs. Diffraction-based methods boast atomic precision but are limited to water that is close to crystalline^11,21,22^ and are insensitive to liquid-phase water dynamics. Linear spectroscopy has revealed distributions of water–water H-bonds in MOFs but similarly lacks time resolution.^23,24^ MD simulations can provide detailed dynamics, which remain to be verified experimentally. Ultrafast time-resolved spectroscopies are not traditionally applied to powders because of optical scatter. However, methods to overcome this issue have been developed in recent years and it is now possible to optically probe the dynamics of molecules in highly scattering solid samples.^25−28^

Here, we reported water H-bonds and dynamics in Ni_2_Cl_2_BTDD (BTDD = bis(1*H*-1,2,3-triazolo[4,5-*b*][4′,5′-*i*])dibenzo[1,4]dioxin) (Figure 1), a promising AWH MOF,^29,30^ using a suite of linear and nonlinear infrared spectroscopies. Ni_2_Cl_2_BTDD boasts a series of linear hexagonal channels with a pore diameter of 2.2 nm, near the “critical diameter” for water adsorption, which allows for high-capacity reversible water uptake over a narrow pore-filling step. We found a similar but stronger H-bond network in the MOF pores than in bulk water. Ultrafast measurements showed that the water network dynamics in Ni_2_Cl_2_BTDD are intermediate between dynamics in bulk water and in other confined systems.^31^ Water in Ni_2_Cl_2_BTDD exhibited an inertial libration that is too fast to be resolved, just like bulk water systems, and constrained slow rotations beyond the lifetime of OD modes, similar to other confined systems. However, water in Ni_2_Cl_2_BTDD displays a picosecond rotation that is constrained in an ∼50° angle. The large angle indicates H-bond rearrangement, highlighting its easiness in Ni_2_Cl_2_BTDD, a key difference from other confined systems.

**Figure 1:**
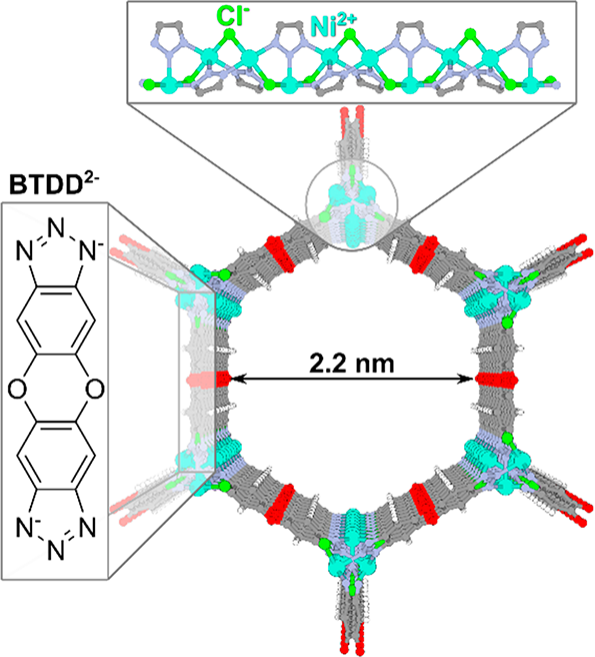
Ni_2_Cl_2_BTDD structure with H-bond acceptors (N, O, and Cl^–^) and open metal sites (Ni^2+^).

Insights into water adsorption in Ni_2_Cl_2_BTDD have already been made using infrared spectroscopy.^12^ However, to obtain further details, it is necessary to reduce spectral congestion, as Ni_2_Cl_2_BTDD and water both have complicated IR spectra. This was accomplished through a combination of isotopic labeling and background subtraction. We mixed D_2_O and H_2_O to obtain a 10% HOD in H_2_O solution. The OD stretch of HOD molecules in H_2_O removes the effects of symmetric and antisymmetric modes and reduces the effects of delocalization and Fermi resonances^32^ that complicate H_2_O spectra.^33^ Subtracting H_2_O/Ni_2_Cl_2_BTDD peaks further simplified the spectra (see Figures S7–S8 in the Supporting Information).

After subtraction, we can detect three separate peaks.^12^ First, there is a sharp high-frequency peak near 2650 cm^–1^, which is a “free water” peak corresponding to OD stretches with no H-bonds^34^ (Figure 2A). Second, there is a main peak around 2500 cm^–1^ that is overlapped with the OD stretches of bulk water. We refer to this central peak as “bulk-like”. Finally, we observed a broad low-frequency shoulder (“strongly bound water”) around 2400 cm^–1^. The “strongly bound water” describes water with high H-bond donor characteristics^35^ (Figure 2D). The Fermi resonance peak^12^ at even lower frequencies disappears in HOD spectra (see Figure S10 in the Supporting Information).

**Figure 2:**
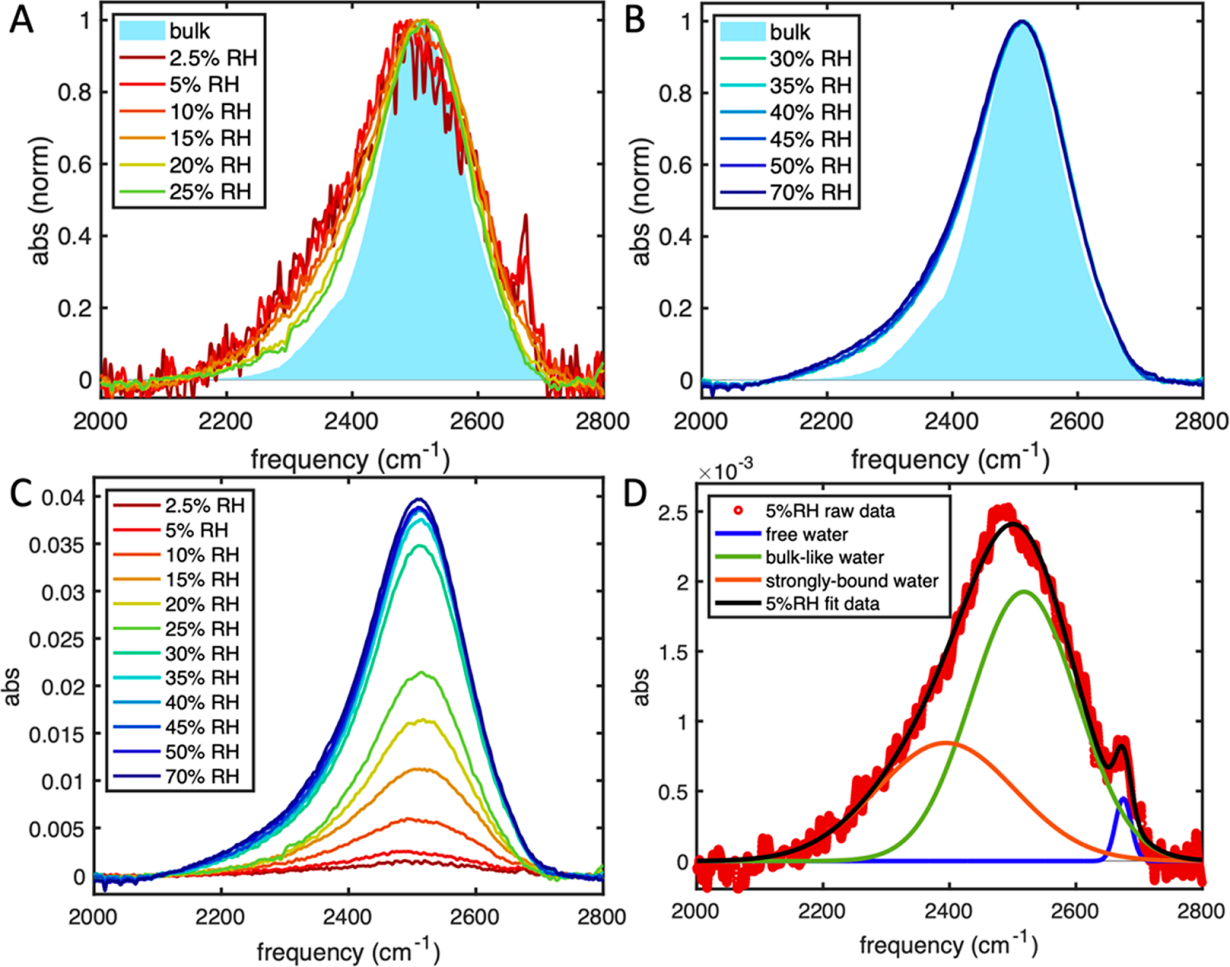
FTIR spectra of HOD in Ni_2_Cl_2_BTDD. Normalized background-subtracted spectra of HOD in Ni_2_Cl_2_BTDD compared to HOD in bulk water (solid filled area) at humidities (A) from 2.5% to 25% RH and (B) from 30% to 70% RH. (C) Raw background-subtracted spectra of HOD in Ni_2_Cl_2_BTDD at humidities from 2.5% to 70% RH. (D) Fitted background-subtracted spectra of HOD in Ni_2_Cl_2_BTDD at 5% RH.

The free, bulk-like, and strongly bound water peaks show dramatic changes in the early stages of pore filling (Figure 2A). The free water peak is only significant below 15% RH, indicating that all water molecules experience H-bonds when the pores are partially or completely filled. This contrasts with FTIR results of some other MOFs, which continue to exhibit the free water peak even when the pores are full.^21,23,36,37^

The strongly bound peak and bulk-like peak, in contrast, remain significant at all water loadings. We assign the strongly bound peak at low water loadings to waters with strong interactions to the framework triazolates and open metal sites. This assignment follows theoretical predictions^31^ and is further supported by the spectra in the fingerprint region, which indicates that the environment around the triazolate group changes most significantly at the lowest water loadings (Figure S9.1). The detailed description of the strongly bound peak at low water loadings is only possible due to the removal of Fermi resonance.^12,36^ The low-frequency end of the OD band decreases in relative intensity as water loading increases, but its absolute absorbance increases with water loading (Figure 2C) and never becomes negligible like the free water peak, which is clear when comparing the MOF HOD spectra to bulk HOD (Figure 2B).

These trends indicate the following filling mechanism: initial water binding occurs at the highly charged open metal and triazolate sites shown in Figure 1. Evidence for this initial binding site is found in large shifts to the triazolate band, which may be due to either direct water–triazolate interactions or changes in the ligand–metal interactions that result from water binding.^38,39^ Additional water molecules then bind to the waters at these charged sites, forming H-bond chains that include most of the pore water at humidities above 15%. The fact that the absorbance of the strongly bound peak increases with water loading indicates strong water–water H-bonds as well. This observation agrees with the water sorption mechanism previously proposed for Ni_2_Cl_2_BTDD.^12,31^ However, FTIR alone reveals few differences between the pore water corresponding to the bulk-like peak and actual bulk water. For a more detailed view of the bulk-like peak, we investigated the ultrafast dynamics of the water molecules.

We collected polarization-selective pump–probe spectroscopy (PSPP) for HOD in Ni_2_Cl_2_BTDD at 25% and 45% RH, below and above the pore-filling step, respectively. The ultrafast laser pulses are tuned specifically centered at the bulk-like peak position, to reveal its dynamics. In PSPP, parallel and perpendicular pump–probe signals are collected as a function of delay time and used to calculate the rotational anisotropy, or Legendre second-order orientational correlation function (*C*_2_(*t*)), which quantifies how fast molecules lose their correlation to original orientations (Figure S11 in the Supporting Information).

The *C*_2_(*t*) dynamics of bulk water and water networks in MOFs show similarities and differences. They decay on similar time scales, but notably, that water dynamics in MOFs decay to an offset, while bulk water relaxes to zero (Figure 3A). Another similarity is that the initial *C*_2_(*t*) of all systems shows a frequency dependence (Figure 3B for 25% RH MOFs, and Figures S13.1 and S13.2 for others). These results are qualitatively similar to previous simulated rotational dynamics of water in Co_2_Cl_2_BTDD.^31^ To extract quantitative information, we fitted the data using the wobbling-in-a-cone model.^40^ The rotational dynamics of wobbling-in-a-cone is described by an initial fast reorientation (<200 fs) constrained within the cone with a semiangle θ_in_, which determines the *C*_2_(*t*) at *t* = 0 (Figure 3E), followed by the molecules rotating in a larger cone leading to a semiangle θ_tot_ (Figure 3E).

**Figure 3:**
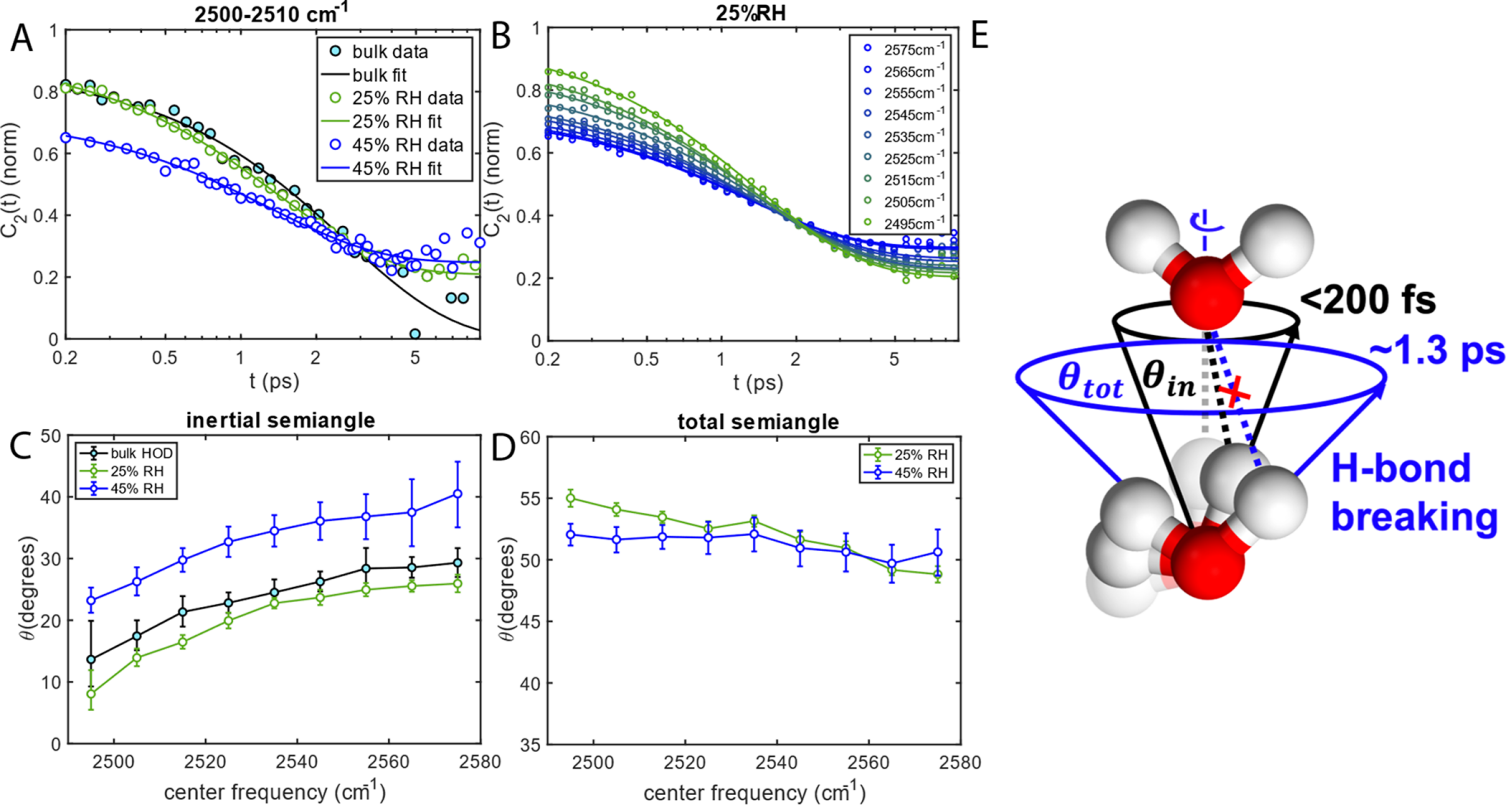
PSPP measurements. (A) Anisotropy dynamics taken from 2500 to 2510 cm^–1^ and (B) anisotropy dynamics at 25% RH taken at each frequency region from 2495 to 2575 cm^–1^. (C) Inertial and (D) total cone semiangles as a function of frequency. Error bars show 95% confidence intervals from fitting. (E) Schemes of wobbling-in-a-cone model. Before wobbling, the hydroxyl group points to the acceptor oxygen with H-bond (gray dashed line). Within <200 fs, the hydroxyl group wobbles within inertial cone semiangle θ_in_ (black cone) along the O–O axis which does not have a large angle to break H-bonds (black dashed line). Within ∼1.3 ps, the hydroxyl group wobbles within the total cone semiangle θ_tot_ (blue cone), large enough to break H-bonds (blue dashed line).

For bulk water, the second rotation is unconstrained (θ_tot_ = 90°) and thus is fitted to a single exponential. The result indicates water fully reoriented with ∼2.4 ps regardless of H-bond intensity (i.e., ω_OH_) while θ_in_ is frequency dependent (Figure 3D). This result agrees with the literature as the strong H-bond leads to tight initial rotations, while the picosecond reorientation describes H-bond rearrangement that only depends on the H-bond acceptor availabilities.^41^

For water H-bond networks in MOFs, at both RHs, their *C*_2_(*t*) values are fitted with a single exponential and a constant, indicating after the initial rotation, there is another constrained reorientation, followed by a slow rotation beyond the OD stretch lifetime. The initial rotation θ_in_ exhibits a frequency dependence similar to that of bulk water, thus depending on H-bond strength (Figure 3C). However, after that, OD wobbles in a confined cone in ∼1.3 ps, different from the unconstrained rotation of bulk water. Similar wobbling-in-a-cone motions have been observed for water in other confined environments.^40,42^ However, at both RHs, θ_tot_ is ∼50°, which is much larger than the θ_tot_ reported in other confined environments, including porous silica and reverse micelles.^40,42^ Indeed, the 50° θ_tot_ is larger than the typical cutoff angle for H-bond definition (∼30°).^43−45^ Thus, it suggests water H-bonds need to rearrange to accommodate this wobbling motion. It is interesting to notice that the wobbling relaxation rate is not quite sensitive to OD frequency, indicating the H-bond network rearrangement does not depend on H-bond strength, similar to bulk water. However, it is less random than bulk water, as it preserves some initial orientational memory. At 25%, the θ_tot_ becomes smaller at higher OH frequency, suggesting a more confined rotation with fewer H-bonds.^35,46−48^ This could suggest a layered structure prior to pore filling, agreeing with prior simulations that predicted *C*_2_(*t*) for water near the pore wall, which is mostly hydrophobic, would have a higher offset than *C*_2_(*t*) for water near the core.^31^ However, after pore filling, θ_tot_ becomes less frequency dependent.

The origin of the picosecond dynamics and cone angle may indicate extra H-bond acceptors in Ni_2_Cl_2_BTDD. Theoretical studies suggested that H-bond rearrangements were limited by the availability of new acceptors.^49^ Chloride ions, the ether, and the triazolate groups could serve as additional acceptors, and FTIR spectra of the strongly bound water peak indicate water with more H-bond interactions.^35^ This mechanism remains to be verified by additional simulations. The fact that the water H-bond network can rearrange on a picosecond time scale in a confined geometry distinguishes Ni_2_Cl_2_BTDD from other confined systems that hinder H-bond rearrangements. The similar rearrangement time scale to bulk water indicates that even after water molecules are trapped inside of these MOFs, it does not cost extra energy to break H-bonds, making reversible desorption feasible.

## Supporting Information Available

Methods, PXRD data, nitrogen isotherm data, FTIR spectra and processing, and PSPP data processing (PDF)

## References

[ref1] DeSantis D.; Mason J. A.; James B. D.; Houchins C.; Long J. R.; Veenstra M. Techno-Economic Analysis of Metal-Organic Frameworks for Hydrogen and Natural Gas Storage. Energy Fuels 2017, 31 (2), 2024–2032. 10.1021/acs.energyfuels.6b02510.

[ref2] Yao Y.; Wang C.; Na J.; Hossain M. S. A.; Yan X.; Zhang H.; Amin M. A.; Qi J.; Yamauchi Y.; Li J. Macroscopic MOF Architectures: Effective Strategies for Practical Application in Water Treatment. Small 2022, 18 (8), 2104387 10.1002/smll.202104387. 34716658

[ref3] Ke F.; Peng C.; Zhang T.; Zhang M.; Zhou C.; Cai H.; Zhu J.; Wan X. Fumarate-Based Metal-Organic Frameworks as a New Platform for Highly Selective Removal of Fluoride from Brick Tea. Sci. Rep. 2018, 8 (1), 939 10.1038/s41598-018-19277-2. 29343778 PMC5772608

[ref4] Rego R. M.; Kuriya G.; Kurkuri M. D.; Kigga M. MOF Based Engineered Materials in Water Remediation: Recent Trends. J. Hazard. Mater. 2021, 403, 123605 10.1016/j.jhazmat.2020.123605. 33264853

[ref5] Kalaj M.; Bentz K. C.; Ayala S. Jr.; Palomba J. M.; Barcus K. S.; Katayama Y.; Cohen S. M. MOF-Polymer Hybrid Materials: From Simple Composites to Tailored Architectures. Chem. Rev. 2020, 120 (16), 8267–8302. 10.1021/acs.chemrev.9b00575. 31895556

[ref6] Lee S. J.; Hann T.; Park S. H. Seawater Desalination Using MOF-Incorporated Cu-Based Alginate Beads without Energy Consumption. ACS Appl. Mater. Interfaces 2020, 12 (14), 16319–16326. 10.1021/acsami.9b22843. 32175730

[ref7] Han X.; Besteiro L. V.; Koh C. S. L.; Lee H. K.; Phang I. Y.; Phan-Quang G. C.; Ng J. Y.; Sim H. Y. F.; Lay C. L.; Govorov A.; Ling X. Y. Intensifying Heat Using MOF-Isolated Graphene for Solar-Driven Seawater Desalination at 98% Solar-to-Thermal Efficiency. Adv. Funct. Mater. 2021, 31 (13), 2008904 10.1002/adfm.202008904.

[ref8] Xu W.; Yaghi O. M. Metal-Organic Frameworks for Water Harvesting from Air, Anywhere, Anytime. ACS Cent. Sci. 2020, 6 (8), 1348–1354. 10.1021/acscentsci.0c00678. 32875075 PMC7453559

[ref9] Shahvari S. Z.; Kalkhorani V. A.; Clark J. D. Performance Evaluation of a Metal Organic Frameworks Based Combined Dehumidification and Indirect Evaporative Cooling System in Different Climates. Int. J. Refrig. 2022, 140, 186–197. 10.1016/j.ijrefrig.2022.05.001.

[ref10] Liu X.; Wang X.; Kapteijn F. Water and Metal-Organic Frameworks: From Interaction toward Utilization. Chem. Rev. 2020, 120 (16), 8303–8377. 10.1021/acs.chemrev.9b00746. 32412734 PMC7453405

[ref11] Hanikel N.; Pei X.; Chheda S.; Lyu H.; Jeong W.; Sauer J.; Gagliardi L.; Yaghi O. M. Evolution of Water Structures in Metal-Organic Frameworks for Improved Atmospheric Water Harvesting. Science 2021, 374 (6566), 454–459. 10.1126/science.abj0890. 34672755

[ref12] Rieth A. J.; Wright A. M.; Skorupskii G.; Mancuso J. L.; Hendon C. H.; Dincǎ M. Record-Setting Sorbents for Reversible Water Uptake by Systematic Anion Exchanges in Metal-Organic Frameworks. J. Am. Chem. Soc. 2019, 141 (35), 13858–13866. 10.1021/jacs.9b06246. 31398286 PMC6748661

[ref13] Ho C.-H.; Valentine M. L.; Chen Z.; Xie H.; Farha O.; Xiong W.; Paesani F. Structure and Thermodynamics of Water Adsorption in NU-1500-Cr. Commun. Chem. 2023, 6 (1), 70 10.1038/s42004-023-00870-0. 37061604 PMC10105746

[ref14] Wagner J. C.; Hunter K. M.; Paesani F.; Xiong W. Water Capture Mechanisms at Zeolitic Imidazolate Framework Interfaces. J. Am. Chem. Soc. 2021, 143 (50), 21189–21194. 10.1021/jacs.1c09097. 34878776

[ref15] Biswas R.; Furtado J.; Bagchi B. Layerwise Decomposition of Water Dynamics in Reverse Micelles: A Simulation Study of Two-Dimensional Infrared Spectrum. J. Chem. Phys. 2013, 139 (14), 144906 10.1063/1.4824446. 24116645

[ref16] Alabarse F. G.; Baptiste B.; Jiménez-Ruiz M.; Coasne B.; Haines J.; Brubach J.-B.; Roy P.; Fischer H. E.; Klotz S.; Bove L. E. Different Water Networks Confined in Unidirectional Hydrophilic Nanopores and Transitions with Temperature. J. Phys. Chem. C 2021, 125 (26), 14378–14393. 10.1021/acs.jpcc.1c01254.

[ref17] Chiashi S.; Saito Y.; Kato T.; Konabe S.; Okada S.; Yamamoto T.; Homma Y. Confinement Effect of Sub-Nanometer Difference on Melting Point of Ice-Nanotubes Measured by Photoluminescence Spectroscopy. ACS Nano 2019, 13 (2), 1177–1182. 10.1021/acsnano.8b06041. 30668902

[ref18] Dokter A. M.; Woutersen S.; Bakker H. J. Anomalous Slowing Down of the Vibrational Relaxation of Liquid Water upon Nanoscale Confinement. Phys. Rev. Lett. 2005, 94 (17), 178301 10.1103/PhysRevLett.94.178301. 15904342

[ref19] Osborne D. G.; Dunbar J. A.; Lapping J. G.; White A. M.; Kubarych K. J. Site-Specific Measurements of Lipid Membrane Interfacial Water Dynamics with Multidimensional Infrared Spectroscopy. J. Phys. Chem. B 2013, 117 (49), 15407–15414. 10.1021/jp4049428. 23931556

[ref20] Huber C. J.; Massari A. M. Characterizing Solvent Dynamics in Nanoscopic Silica Sol-Gel Glass Pores by 2D-IR Spectroscopy of an Intrinsic Vibrational Probe. J. Phys. Chem. C 2014, 118 (44), 25567–25578. 10.1021/jp508389u.

[ref21] Ichii T.; Arikawa T.; Omoto K.; Hosono N.; Sato H.; Kitagawa S.; Tanaka K. Observation of an Exotic State of Water in the Hydrophilic Nanospace of Porous Coordination Polymers. Commun. Chem. 2020, 3 (1), 1–6. 10.1038/s42004-020-0262-9. 36703440 PMC9814769

[ref22] Bae J.; Park S. H.; Moon D.; Jeong N. C. Crystalline Hydrogen Bonding of Water Molecules Confined in a Metal-Organic Framework. Commun. Chem. 2022, 5 (1), 1–10. 10.1038/s42004-022-00666-8. 36697686 PMC9814150

[ref23] Gao J.; Fei S.; Ho Y.-L.; Matsuda R.; Daiguji H.; Delaunay J.-J. Water Confined in MIL-101(Cr): Unique Sorption-Desorption Behaviors Revealed by Diffuse Reflectance Infrared Spectroscopy and Molecular Dynamics Simulation. J. Phys. Chem. C 2021, 125 (32), 17786–17795. 10.1021/acs.jpcc.1c03351.

[ref24] Hiraoka T.; Shigeto S. Interactions of Water Confined in a Metal-Organic Framework as Studied by a Combined Approach of Raman, FTIR, and IR Electroabsorption Spectroscopies and Multivariate Curve Resolution Analysis. Phys. Chem. Chem. Phys. 2020, 22 (32), 17798–17806. 10.1039/D0CP02958K. 32609125

[ref25] Hack J. H.; Dombrowski J. P.; Ma X.; Chen Y.; Lewis N. H. C.; Carpenter W. B.; Li C.; Voth G. A.; Kung H. H.; Tokmakoff A. Structural Characterization of Protonated Water Clusters Confined in HZSM-5 Zeolites. J. Am. Chem. Soc. 2021, 143 (27), 10203–10213. 10.1021/jacs.1c03205. 34210123

[ref26] Yan C.; Nishida J.; Yuan R.; Fayer M. D. Water of Hydration Dynamics in Minerals Gypsum and Bassanite: Ultrafast 2D IR Spectroscopy of Rocks. J. Am. Chem. Soc. 2016, 138 (30), 9694–9703. 10.1021/jacs.6b05589. 27385320

[ref27] Nishida J.; Fayer M. D. Guest Hydrogen Bond Dynamics and Interactions in the Metal-Organic Framework MIL-53(Al) Measured with Ultrafast Infrared Spectroscopy. J. Phys. Chem. C 2017, 121 (21), 11880–11890. 10.1021/acs.jpcc.7b02458.

[ref28] Nishida J.; Tamimi A.; Fei H.; Pullen S.; Ott S.; Cohen S. M.; Fayer M. D. Structural Dynamics inside a Functionalized Metal-Organic Framework Probed by Ultrafast 2D IR Spectroscopy. Proc. Natl. Acad. Sci. U. S. A. 2014, 111 (52), 18442–18447. 10.1073/pnas.1422194112. 25512539 PMC4284562

[ref29] Rieth A. J.; Yang S.; Wang E. N.; Dincǎ M. Record Atmospheric Fresh Water Capture and Heat Transfer with a Material Operating at the Water Uptake Reversibility Limit. ACS Cent. Sci. 2017, 3 (6), 668–672. 10.1021/acscentsci.7b00186. 28691080 PMC5492259

[ref30] Bagi S.; Wright A. M.; Oppenheim J.; Dincǎ M.; Román-Leshkov Y. Accelerated Synthesis of a Ni2Cl2(BTDD) Metal-Organic Framework in a Continuous Flow Reactor for Atmospheric Water Capture. ACS Sustain. Chem. Eng. 2021, 9 (11), 3996–4003. 10.1021/acssuschemeng.0c07055.

[ref31] Rieth A. J.; Hunter K. M.; Dincǎ M.; Paesani F. Hydrogen Bonding Structure of Confined Water Templated by a Metal-Organic Framework with Open Metal Sites. Nat. Commun. 2019, 10 (1), 4771 10.1038/s41467-019-12751-z. 31628319 PMC6802106

[ref32] Woutersen S.; Bakker H. J. Resonant Intermolecular Transfer of Vibrational Energy in Liquid Water. Nature 1999, 402 (6761), 507–509. 10.1038/990058.

[ref33] De Marco L.; Ramasesha K.; Tokmakoff A. Experimental Evidence of Fermi Resonances in Isotopically Dilute Water from Ultrafast Broadband IR Spectroscopy. J. Phys. Chem. B 2013, 117 (49), 15319–15327. 10.1021/jp4034613. 23638966

[ref34] Dalla Bernardina S.; Paineau E.; Brubach J.-B.; Judeinstein P.; Rouzière S.; Launois P.; Roy P. Water in Carbon Nanotubes: The Peculiar Hydrogen Bond Network Revealed by Infrared Spectroscopy. J. Am. Chem. Soc. 2016, 138 (33), 10437–10443. 10.1021/jacs.6b02635. 27455124

[ref35] Auer B.; Kumar R.; Schmidt J. R.; Skinner J. L. Hydrogen Bonding and Raman, IR, and 2D-IR Spectroscopy of Dilute HOD in Liquid D2O. Proc. Natl. Acad. Sci. U. S. A. 2007, 104 (36), 14215–14220. 10.1073/pnas.0701482104. 17576923 PMC1964876

[ref36] Hunter K. M.; Wagner J. C.; Kalaj M.; Cohen S. M.; Xiong W.; Paesani F. Simulation Meets Experiment: Unraveling the Properties of Water in Metal-Organic Frameworks through Vibrational Spectroscopy. J. Phys. Chem. C 2021, 125 (22), 12451–12460. 10.1021/acs.jpcc.1c03145.

[ref37] Lu Z.; Duan J.; Du L.; Liu Q. M.; Schweitzer N. T.; Hupp J. Incorporation of Free Halide Ions Stabilizes Metal-Organic Frameworks (MOFs) against Pore Collapse and Renders Large-Pore Zr-MOFs Functional for Water Harvesting. J. Mater. Chem. A 2022, 10 (12), 6442–6447. 10.1039/D1TA10217F.

[ref38] Andreeva A. B.; Le K. N.; Kadota K.; Horike S.; Hendon C. H.; Brozek C. K. Cooperativity and Metal-Linker Dynamics in Spin Crossover Framework Fe(1,2,3-Triazolate)2. Chem. Mater. 2021, 33 (21), 8534–8545. 10.1021/acs.chemmater.1c03143.

[ref39] Fabrizio K.; Andreeva A. B.; Kadota K.; Morris A. J.; Brozek C. K. Guest-Dependent Bond Flexibility in UiO-66, a “Stable” MOF. Chem. Commun. 2023, 59 (10), 1309–1312. 10.1039/D2CC05895B. 36636868

[ref40] Tan H.-S.; Piletic I. R.; Fayer M. D. Orientational Dynamics of Water Confined on a Nanometer Length Scale in Reverse Micelles. J. Chem. Phys. 2005, 122 (17), 174501 10.1063/1.1883605. 15910039

[ref41] Laage D.; Hynes J. T. Do More Strongly Hydrogen-Bonded Water Molecules Reorient More Slowly ?. Chem. Phys. Lett. 2006, 433 (1), 80–85. 10.1016/j.cplett.2006.11.035.

[ref42] Yamada S. A.; Hung S. T.; Thompson W. H.; Fayer M. D. Effects of Pore Size on Water Dynamics in Mesoporous Silica. J. Chem. Phys. 2020, 152 (15), 154704 10.1063/1.5145326. 32321257

[ref43] Lawrence C. P.; Skinner J. L. Vibrational Spectroscopy of HOD in Liquid D2O. III. Spectral Diffusion, and Hydrogen-Bonding and Rotational Dynamics. J. Chem. Phys. 2003, 118 (1), 264–272. 10.1063/1.1525802.

[ref44] Møller K. B.; Rey R.; Hynes J. T. Hydrogen Bond Dynamics in Water and Ultrafast Infrared Spectroscopy: A Theoretical Study. J. Phys. Chem. A 2004, 108 (7), 1275–1289. 10.1021/jp035935r.

[ref45] Luzar A.; Chandler D. Structure and Hydrogen Bond Dynamics of Water-Dimethyl Sulfoxide Mixtures by Computer Simulations. J. Chem. Phys. 1993, 98 (10), 8160–8173. 10.1063/1.464521.

[ref46] Kumar R.; Schmidt J. R.; Skinner J. L. Hydrogen Bonding Definitions and Dynamics in Liquid Water. J. Chem. Phys. 2007, 126 (20), 204107 10.1063/1.2742385. 17552754

[ref47] Tainter C. J.; Skinner J. L. The Water Hexamer: Three-Body Interactions, Structures, Energetics, and OH-Stretch Spectroscopy at Finite Temperature. J. Chem. Phys. 2012, 137 (10), 104304 10.1063/1.4746157. 22979856

[ref48] Tainter C. J.; Ni Y.; Shi L.; Skinner J. L. Hydrogen Bonding and OH-Stretch Spectroscopy in Water: Hexamer (Cage), Liquid Surface, Liquid, and Ice. J. Phys. Chem. Lett. 2013, 4 (1), 12–17. 10.1021/jz301780k. 26291204

[ref49] Laage D.; Hynes J. T. A Molecular Jump Mechanism of Water Reorientation. Science 2006, 311 (5762), 832–835. 10.1126/science.1122154. 16439623

