# Supplementary material for: Ultrafast Water H-Bond Rearrangement in a Metal–Organic Framework Probed by Femtosecond Time-Resolved Infrared Spectroscopy
Source: J Am Chem Soc. 2023 May 18;145(21):11482–7. doi: 10.1021/jacs.3c01728 (PMC10236489; doi:10.1021/jacs.3c01728)
Supplement: Supplementary file 1 — ja3c01728_si_001.pdf [file ja3c01728_si_001.pdf]

Ultrafast Water H-bond Rearrangement in a Metal Organic Framework Probed by Femtosecond Time-Resolved Infrared Spectroscopy

Mason L. Valentine[a§], Guoxin Yin[b§], Julius J. Oppenheim[c], Mircea Dincă[c], Wei Xiong[a,b]*

[a]*Department of Chemistry and Biochemistry, University of California San Diego, La Jolla, California 92093, United States;* orcid.org/0000-0001-5412-9772
[b]*Materials Science and Engineering, University of California San Diego, La Jolla, California 92093, United States;*
[c]*Department of Chemistry, Massachusetts Institute of Technology, 77 Massachusetts Avenue Cambridge, Massachusetts 02139, United States;*

CONTENTS

Contents 1
S1: General Information 3
S2: Synthetic Methods 6
S3: Optical Setup 7
S4: Powder X-ray Diffraction Data 9
S5: Nitrogen Isotherm Data 10
S6: Raw FTIR Spectra 11
S7: O-D Stretch Baseline Subtraction 13
S8: O-D Stretch Fits 14
S9: Fingerprint Region FTIR 16
S10: O-H Stretching Region FTIR 18
S11: Population Decay and Heating Effect Removal 19
S12: Förster Energy Transfer Simulations 25
S13: Anisotropy Decay 26
S14: References 30

S1: GENERAL INFORMATION

Materials and Methods. $NiCl_2$•$6H_2O$ (Strem Chemicals), HCl (32-35%, BDC – VWR Analytic), methanol (99.9%, VWR), and N,N-dimethylformamide (99.8%, Millipore) were used as received. H_2BTDD was synthesized according to literature.[1] Deuterium oxide (D 99.9%) was purchased from Cambridge Isotope Labs. Ultrapure water (18 MΩcm^{-1}) was obtained from a Sartorius Arium Pro system. 25.4 cm diameter Calcium fluoride windows were purchased from Crystran, Inc. Fluorolube was purchased from Sigma Aldrich.

Powder X-ray diffraction (PXRD) patterns were recorded with a Bruker D8 Advance II diffractometer equipped with Ni-filtered CuKa radiation (Ka_1 = 1.5406 Å, Ka_2 = 1.5444 Å, Ka_1/Ka_2 = 0.5). The tube voltage and current were 40 kV and 40 mA, respectively. The measurement was performed in a Bragg-Brentano geometry. The samples were prepared by placing a thin layer of material on top of a zero-background silicon crystal plate.

Nitrogen adsorption isotherms were measured by a volumetric method using a Micromeritics ASAP 2020 Plus gas sorption analyzer. In an argon filled glovebox, 39.8 mg of pre-activated sample (100 °C) were transferred into a pre-weighed analysis tube, capped with a Micromeritics TranSeal, and transferred to the analysis port of the gas sorption analyzer. Free space correction measurements were performed using ultra-high purity He gas (UHP grade 5, 99.999% pure). Nitrogen isotherms were measured using UHP grade nitrogen and using a liquid nitrogen bath at 77 K. Oil-free vacuum pumps were used to prevent contamination of sample or feed gases.

Infrared Sample Preparation: Ni_2Cl_2BTDD was weighted into plastic centrifuge tubes with ultrapure water at a concentration of 40mg/mL and suspended via sonication for 10 minutes. A 20uL aliquot of the resulting slurry was then dropcast onto the center of a CaF_2 window and the water was allowed to evaporate at ambient pressure and temperature overnight. FTIR samples were placed in a lens tube modified to include a gas inlet and sealed on the other end with a clean CaF_2 window, then transferred directly to the FTIR

instrument for experiments. 10% HOD solutions were made by diluting 5mL D_2O with ultrapure water in a 100mL volumetric flask.

Polarization-selective pump-probe (PSPP) samples were hydrated with 10% HOD, then converted into oil mulls to reduce optical scatter prior to experiments. The samples were placed in a glove bag filled with a stream of dry N_2 combined with a stream of N_2 bubbled through a 10% HOD solution. The relative humidity of the combined stream was monitored using a data logger (HOBO) calibrated using saturated solutions of $MgCl_2$, K_2CO_3, and NaCl and controlled using 2 valves to vary the flow rates of the dry N_2 and 10% HOD-saturated N_2 streams. To hydrate, the humidity was increased to >60% RH for a minimum of 20 minutes, and to dehydrate, the humidity was decreased to <5% RH for a minimum of 20 minutes. 20 minute equilibration times were used in previous infrared studies of Ni2Cl2BTDD,[1] and we observed that our samples equilibrated to each relative humidity in a similar amount of time. To load the pores with 10% HOD, we equilibrated the pores below 5% RH, then above 60% RH with 10% HOD vapor three times. After H/D exchange, the humidity was adjusted to the sample's set relative humidity for a minimum of 20 minutes before placing a drop of Fluorolube onto the sample and pressing another window down to seal the sample. The top window was then gently rotated back and forth to shear the sample until the scatter was below 0.5 absorbance units and the optical density of the O-D stretching mode was between 0.1 and 0.5 absorbance units as observed by FTIR. To achieve a sufficiently high optical density below the pore-filling step, it was necessary to dropcast the MOF slurry twice onto both CaF_2 windows, yielding samples roughly 4 times as thick as the FTIR samples and high RH samples.

FTIR: Spectra were recorded using a Thermo Fisher Nicolet IS10 spectrometer. Samples were held in a home-built gas cell made by drilling holes in a lens tube and placing CaF_2 windows at the ends. The humidity in the gas cell was controlled using the N_2 and water vapor streams described in the sample preparation section, and a 20-minute equilibration time was used at each humidity before acquiring the reported spectrum. In order to obtain an acceptable signal-to-noise ratio we used 64 scans with a frequency resolution of 4 cm^{-1} over a frequency range from 1110 to 4000 cm^{-1}. H_2O spectra were collected first, then

to equilibrate HOD in the pores, we performed an *in situ* H/D exchange with 3 hydration/dehydration cycles of 10% HOD and 20 minute equilibration times. After H/D exchange, we repeated the humidity-dependent experiment with 10% HOD instead of neat water. The HOD and H_2O spectra were both collected starting with the highest humidity, then the humidity was decreased throughout the experiment. After collecting the spectra of 10% HOD and H_2O in the MOF pores, we collected spectra of HOD vapor in an otherwise empty cell for atmospheric correction. The raw FTIR spectra before background subtraction are shown in Supporting Information S6. The H_2O spectra were subtracted from the HOD spectra, then a linear baseline correction was applied to the background-subtracted spectra in the O-D stretching region, which is shown in Supporting Information S7.

S2: SYNTHETIC METHODS

Ni_2Cl_2BTDD was synthesized according to literature.[1] 202.95 mg H_2BTDD (0.76 mmol, 1 eq.) were dissolved in 200 mL N,N-dimethylformamide (DMF) in a 500 mL jar by heating to 140 °C. The clear solution was then cooled to room temperature. In a 2 L jar, 394.58 mg $NiCl_2 \bullet 6H_2O$ (1.66 mmol, 2.18 eq.) were dissolved in a solution of 200 mL methanol and 128 mL concentrated hydrochloric acid. The solution of H_2BTDD was added to the solution of nickel chloride, and then heated to 100 °C in an oven for 2 days. After this time, the reaction mixture was allowed to cool to room temperature and the solids were collected by filtration. The solids were washed five times with 30 mL DMF and then five times with 30 mL MeOH. The solids were activated under dynamic vacuum at 150 °C for 24 hours. The yield was 305 mg of a green colored solid.

S3: OPTICAL SETUP

The setup scheme is shown in **Figure. S3.1**. 800 nm laser pulses (~35 fs, ~5 W, 1 kHz) generated by an

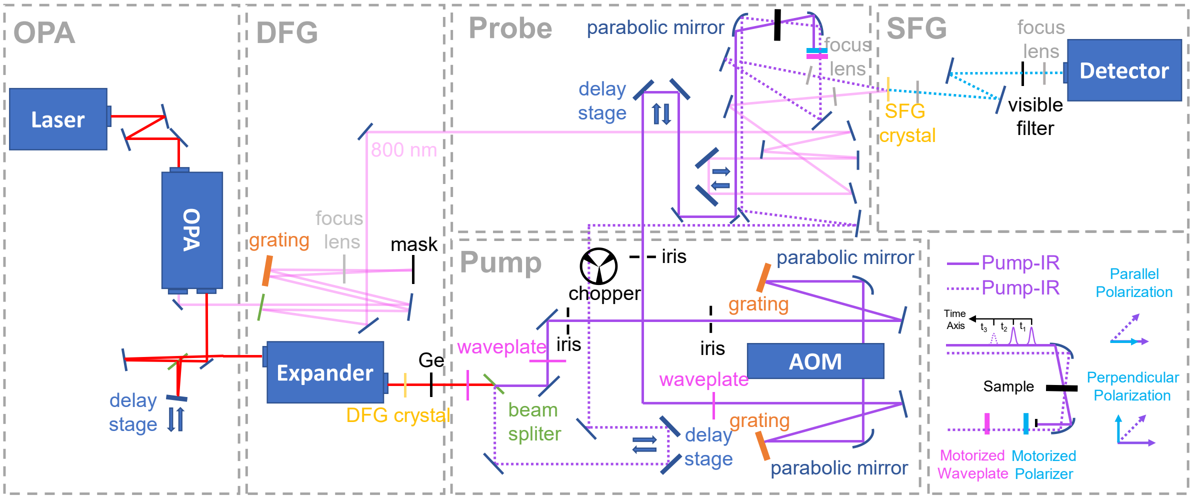

Figure S3.1: Pump-probe setup; Inset on the bottom right: motorized waveplate and polarizer after sample for anisotropy measurement.

ultrafast Ti:Sapphire regenerative amplifier (Astrella, Coherent) are sent into an optical parametric amplifier (OPA) (TOPAS, LightConversion) which outputs tunable near-IR pulses. The near-IR pulses are converted to mid-IR pulses through a difference frequency generation (DFG) process by a type II $AgGaS_2$ crystal. After DFG, a CaF_2 wedge splits the mid-IR pulse into two parts: 95% of the pulse is transmitted and sent into a Ge Acoustic Optical Modulator based mid-IR pulse shaper (QuickShape, PhaseTech) and then chopped by shaper, which form the pump beam arm; the 5% reflected is the probe beam. Both pump and probe are focused by a parabolic mirror (f = 10 cm) and overlapped spatially at the sample. The output signal is collimated by another parabolic mirror (f = 10 cm) and upconverted to visible wavelengths by an 800 nm beam at a $LiNbO_3$ crystal. The 800 nm beam, which comes out of the OPA, passes through an 800 nm pulse shaper which narrows its spectrum in the frequency domain (center wavelength of 794.5 nm and the FWHM of 0.5 nm or 7.9 cm^{-1}).

The polarization-selective pump-probe (PSPP) measurement is achieved using motorized waveplate and polarizer after sample as shown in the inset of **Figure. S3.1**. The angle between the polarizations of the pump and probe pulses is 45 degrees. For parallel polarization pump-probe measurements, the motorized polarizer after the sample is set to only transmit light with a polarization parallel to that of pump pulse. For

perpendicular polarization measurements, the polarizer is rotated to only transmit light with a polarization perpendicular to the pump pulse. The motorized waveplate after the polarizer is used to maintain a constant signal polarization right before the $LiNbO_3$ crystal used for upconversion. Every pump-probe scan requires 10-20 min at any polarization, so, we changed the polarization after every scan and performed hundreds of repeated scans to reduce the effects of drift and obtain a high signal-to-noise ratio.

The roughness of MOF sample induced strong pump scatter including pure pump scatter and pump-probe interference scatter. We used the pulse shaper to generate 8-frame pump sequences by phase cycling, and chopped the probe pulse in 500 Hz. Either odd or even frame in 8-frame was standard 4-frame phase cycling to remove the interference scatter by data processing. But when pump pulses were in even frames, the probe would be chopped, so pure pump scatter would be measured and removed by data processing as well. This combination of 4-frame phase cycling and probe pulse chopping, i.e. 8-frame phase cycling, can effectively remove both the pure pump scatter and interference scatter in highly scattering samples.[2]

S4: POWDER X-RAY DIFFRACTION DATA

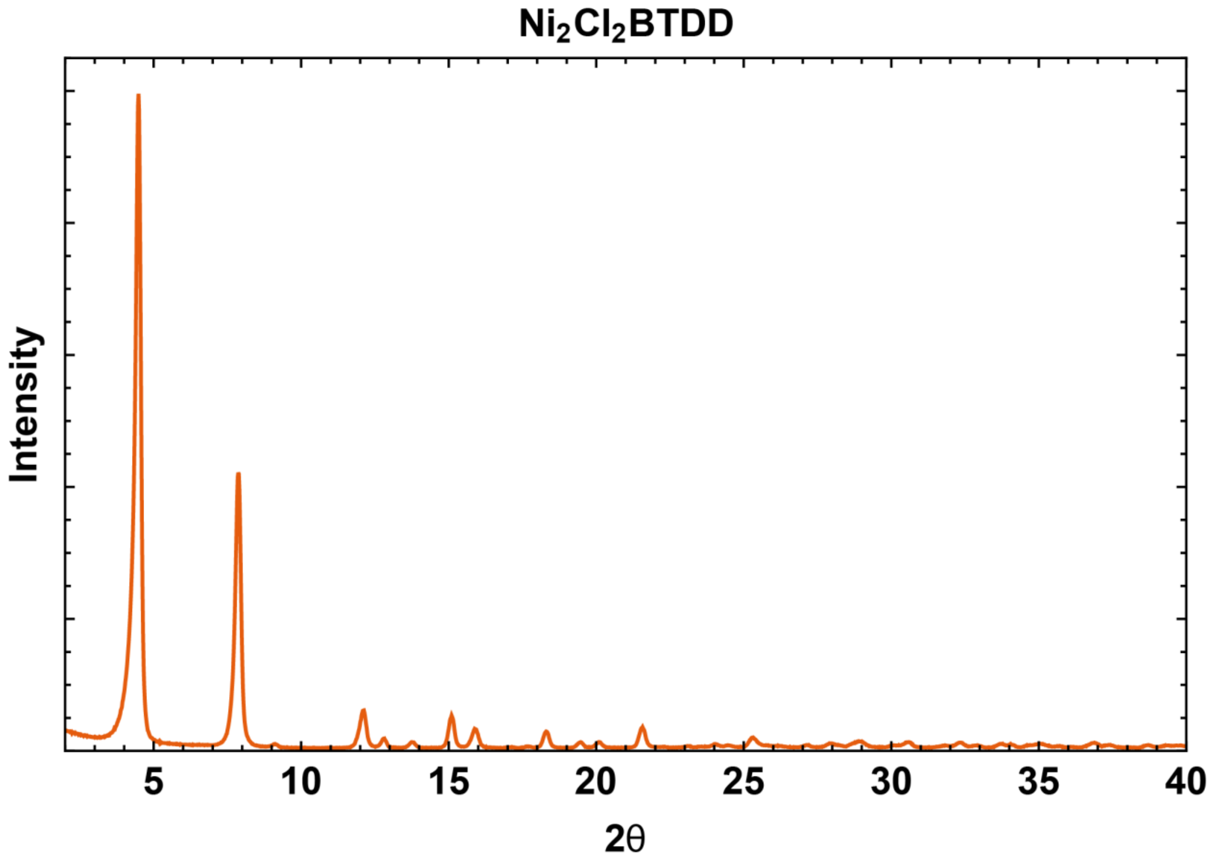

Figure S4.1: PXRD data for Ni_2Cl_2BTDD

S5: NITROGEN ISOTHERM DATA

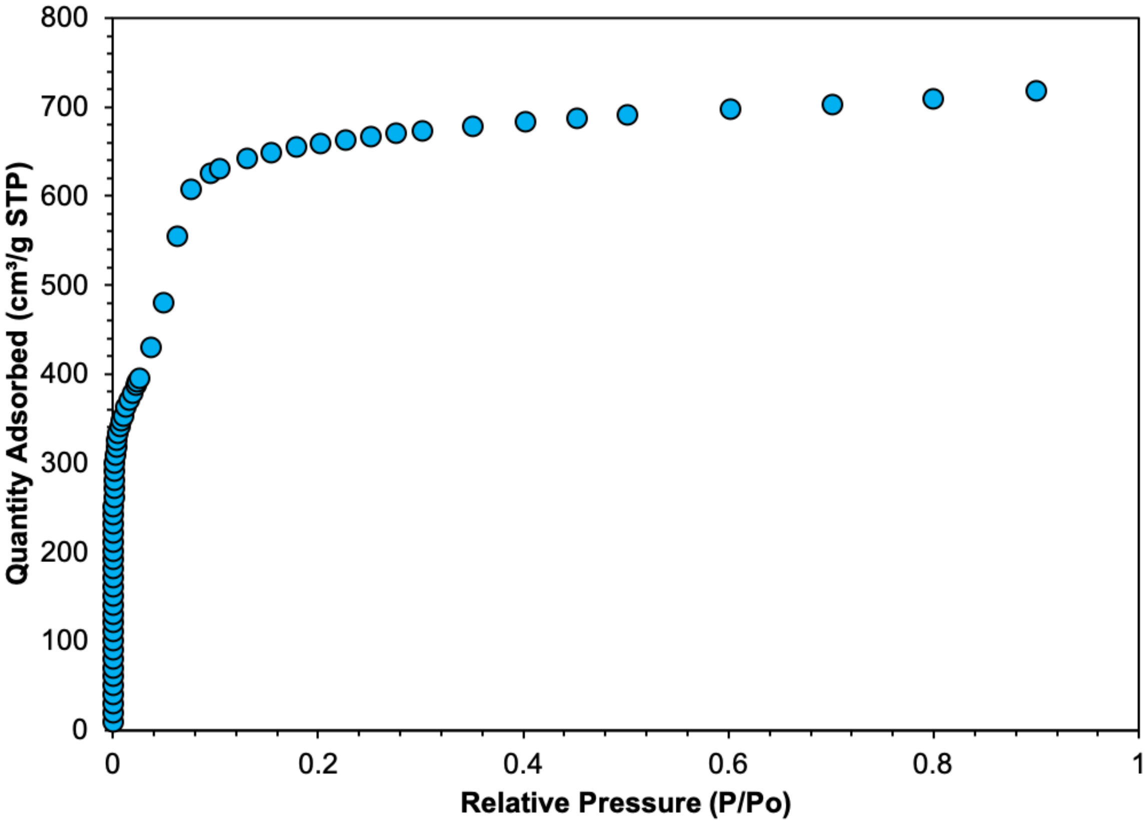

Figure S5.1: N_2 adsorption isotherm for Ni_2Cl_2BTDD.

S6: RAW FTIR SPECTRA

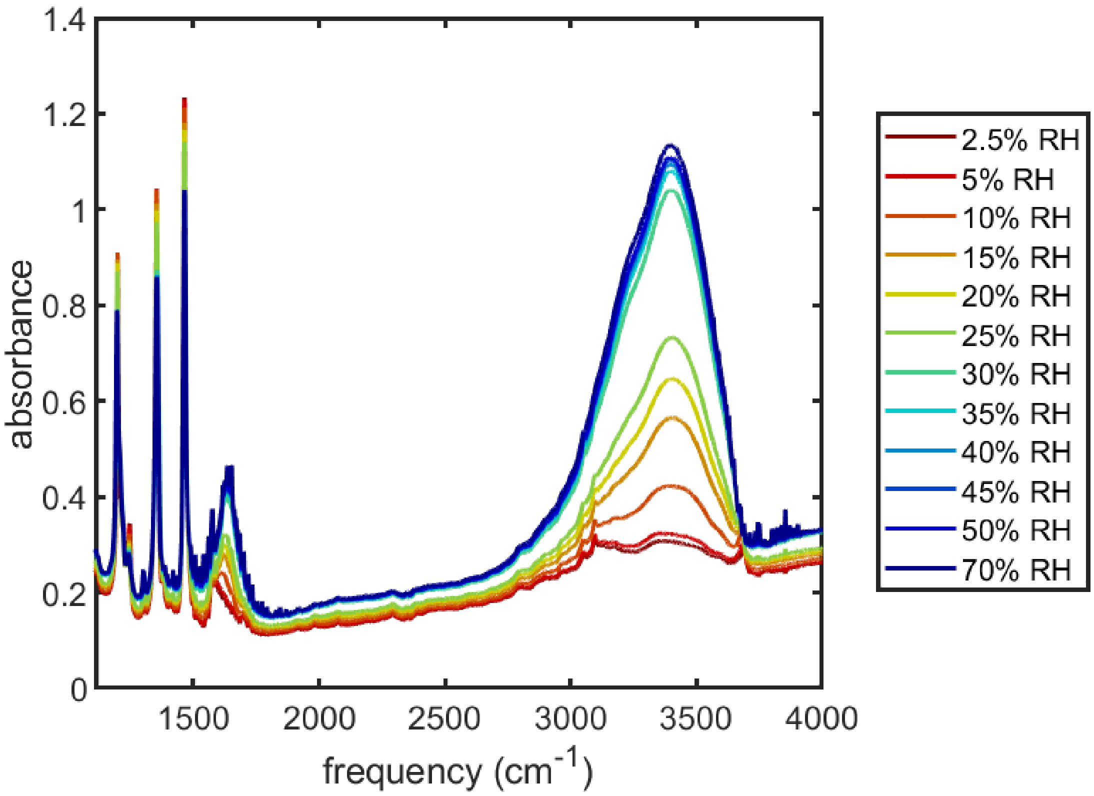

Figure S6.1: Raw FTIR spectra of Ni_2Cl_2BTDD and H2O at humidities from 2.5% RH to 70% RH.

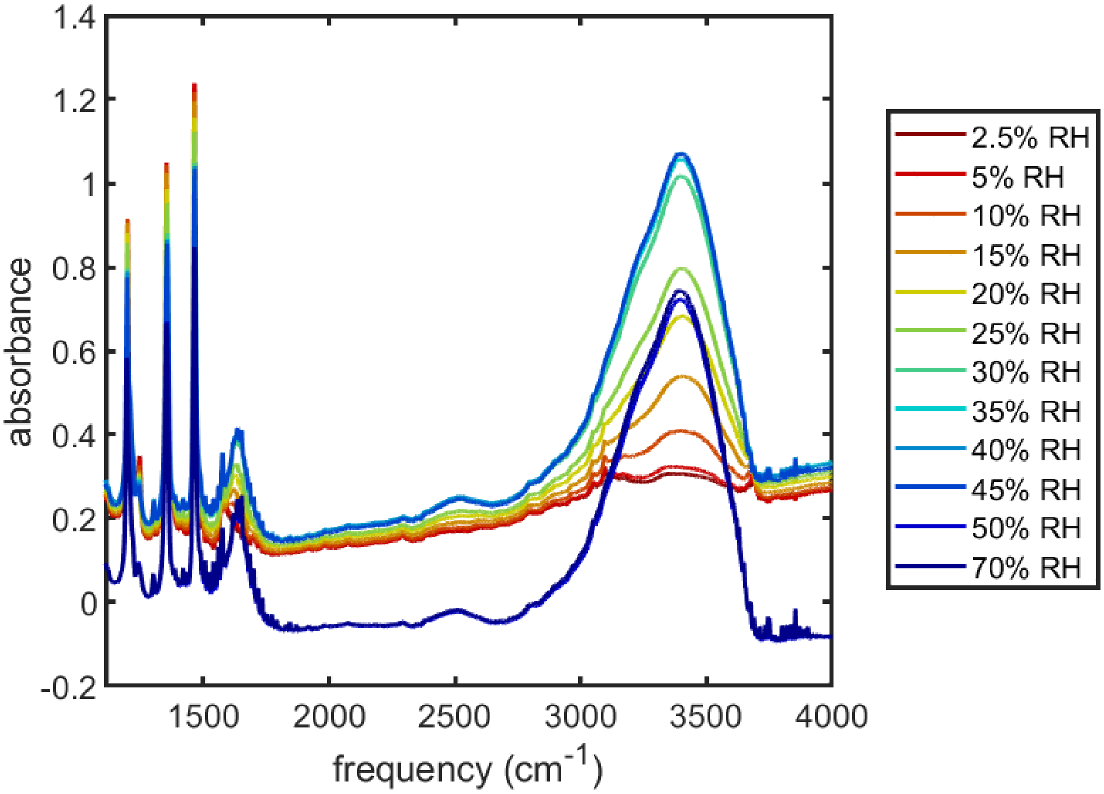

Figure S6.2: Raw FTIR spectra of Ni_2Cl_2BTDD and 10% HOD in H_2O at humidities from 2.5% RH to 70% RH.

S7: O-D STRETCH BASELINE SUBTRACTION

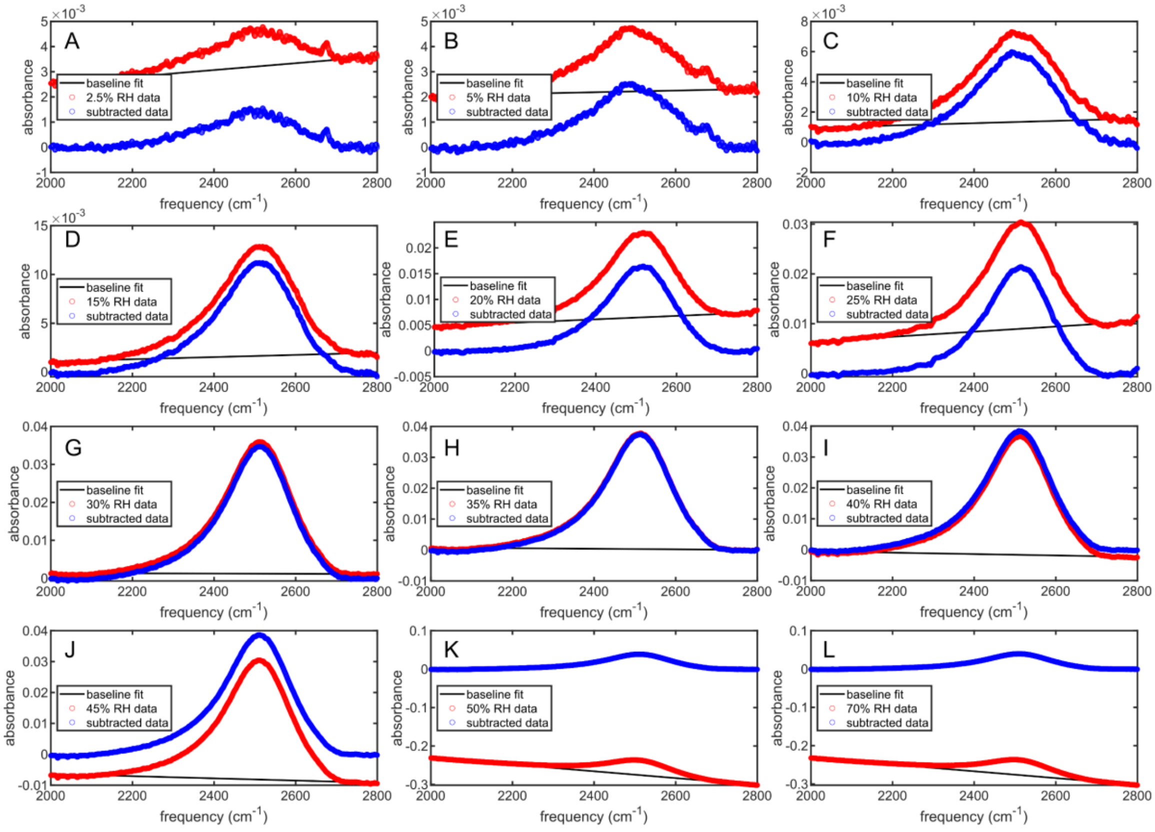

Figure S7.1: Background-subtracted O-D stretch region before and after linear baseline subtraction for (A) 2.5% RH (B) 5% RH (C) 10% RH (D) 15% RH (E) 20% RH (F) 25% RH (G) 30% RH (H) 35% RH (I) 40% RH (J) 45% RH (K) 50% RH, and (L) 70% RH. Data before baseline subtraction is shown in red, data after baseline subtraction is shown in blue, and the baseline is shown in black.

S8: O-D STRETCH FITS

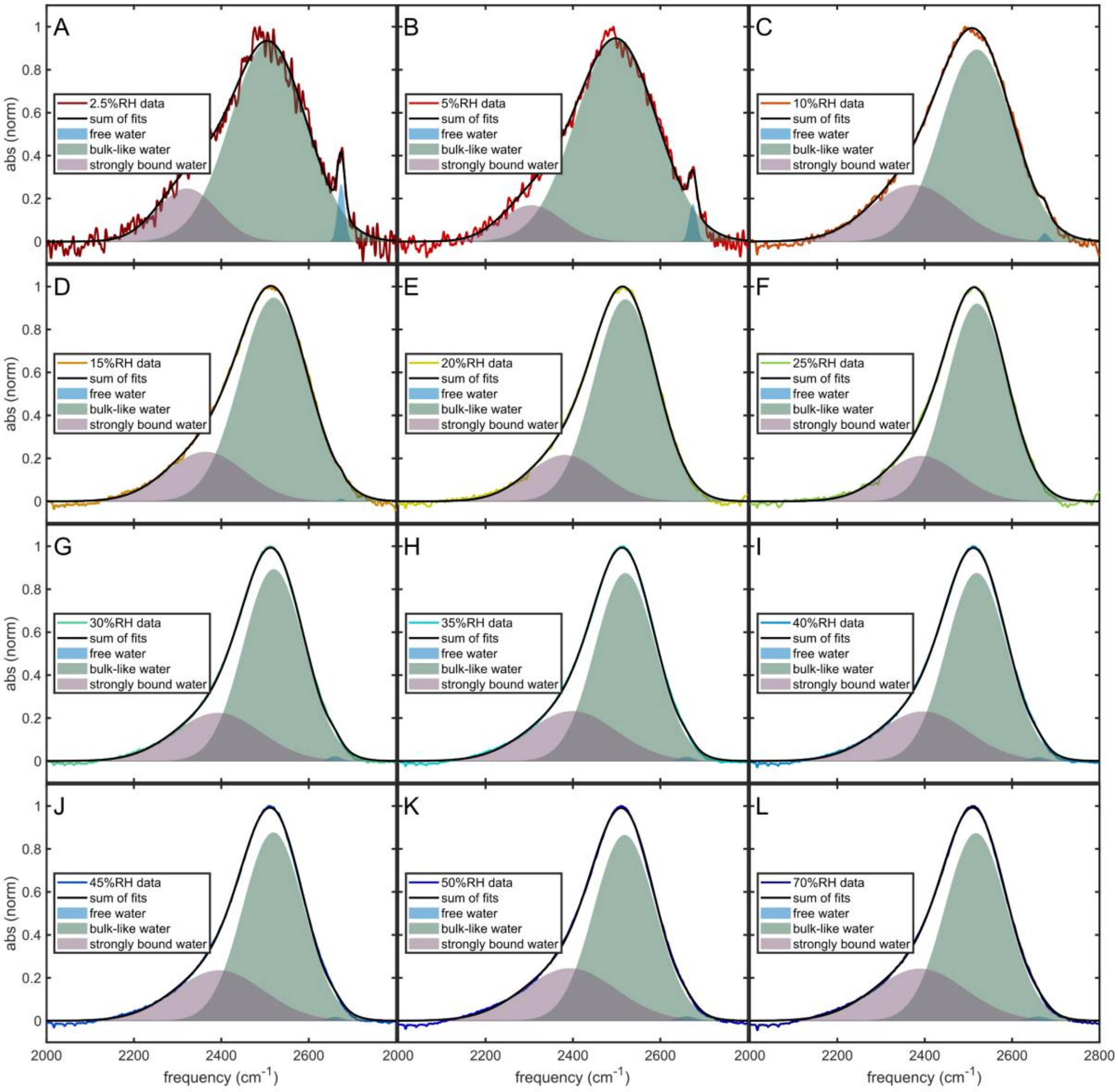

Figure S8.1: Gaussian fits to the O-D stretching band of 10% HOD in Ni2Cl2BTDD recorded at (A) 2.5% RH (B) 5% RH (C) 10% RH (D) 15% RH (E) 20% RH (F) 25% RH (G) 30% RH (H) 35% RH (I) 40% RH (J) 45% RH (K) 50% RH, and (L) 70% RH.

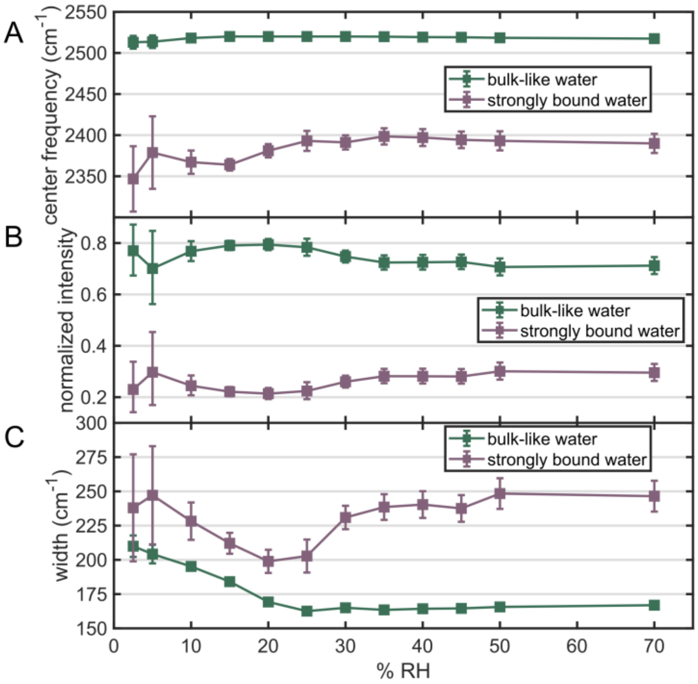

Figure S8.2: Humidity-dependent trends in peak properties. (A) The center frequencies of the bulk-like (green) and strongly bound (magenta) water peaks as a function of humidity. (B) The relative intensities of the bulk-like (green) and strongly bound (magenta) water peaks as a function of humidity. (C) The widths (full widths at half maximum) of the bulk-like (green) and strongly bound (magenta) water peaks as a function of humidity. Peak properties are highly dependent on water loading.

S9: FINGERPRINT REGION FTIR

Ni_2Cl_2BTDD exhibits three prominent bands between 1150 cm^{-1} and 1500 cm^{-1}. One band, centered around 1200 cm^{-1} and composed of three overlapping peaks, is similar to a set of temperature-sensitive triazolate ring modes previously observed in triazolate MOFs with a variety of metals.[3] The other two prominent bands, centered around 1350cm^{-1} and 1475 cm^{-1}, were assigned to ether C-O and aromatic C-C stretches, respectively. All bands experienced a discontinuous broadening and decrease in maximum absorbance around the pore-filling step, indicating that they are all sensitive to the presence of water. To analyze changes to the peak shapes as a function of water loading, we calculated the bands' centers of mass, $<\omega>$, via the following equation.

$$\langle\omega\rangle = \frac{\sum[A(\omega_i)\cdot\omega_i]}{\sum A(\omega_i)} \tag{S9.1}$$

Where ω_i are the frequencies within the band and $A(\omega_i)$ is the absorbance as a function of frequency. This approach is identical to calculating the mean of a histogram. We observed that the triazolate peak shifted most at low humidities, while the C-C and C-O stretching bands experienced their largest shifts near the pore-filling step (Figure S9.1B).

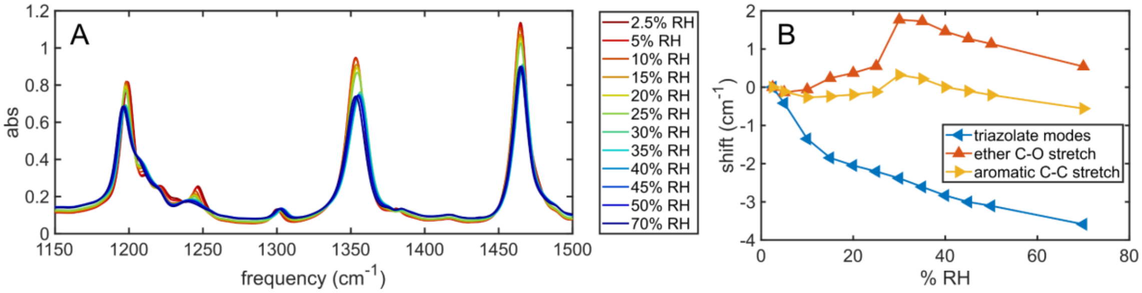

Figure S9.1: FTIR spectra of Ni_2Cl_2BTDD and H_2O in the fingerprint region. (A) all spectra after linear baseline subtraction at humidities from 2.5% RH (dark red) to 70% RH (dark blue). The triazolate ring mode peaks are centered around 1200 cm^{-1}, the ether C-O stretch peaks are near 1350 cm^{-1}, and the aromatic C-C stretching modes are around 1475 cm^{-1} (B) Spectral shifts for each of the three strong MOF absorption peaks. Shifts were calculated using changes in peaks' centers of mass. The triazolate modes shift most significantly at low water loadings, while the ether C-O and aromatic C-C stretches shift mostly near the pore-filling humidity.

S10: O-H STRETCHING REGION FTIR

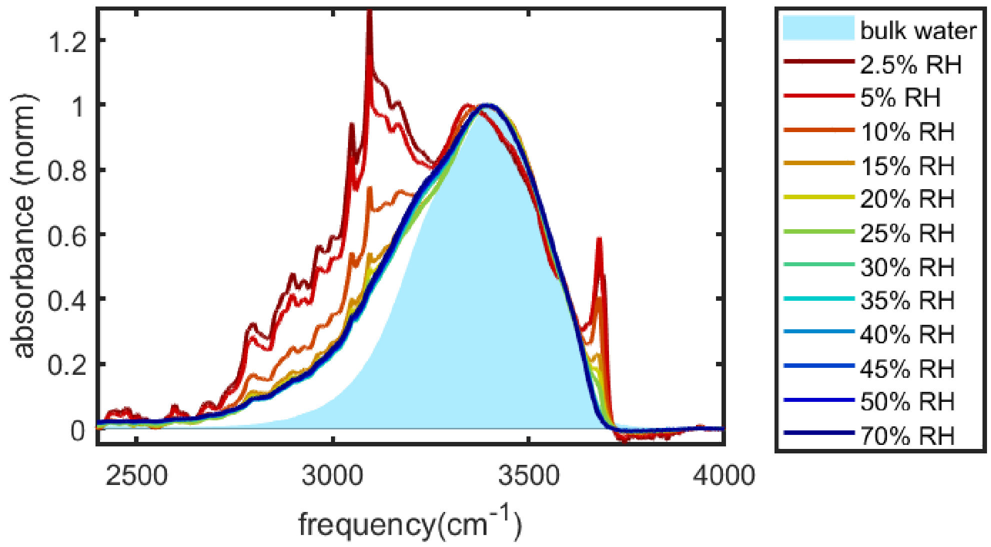

Figure S10.1: FTIR spectra of H_2O in Ni_2Cl_2BTDD at humidities from 2.5% RH to 70% RH in the O-H stretching region. The overall trends match those observed in the O-D stretching region, but there is interference from C-H modes and the H-O-H bending overtone obscuring the low-frequency side of the O-H stretching band.

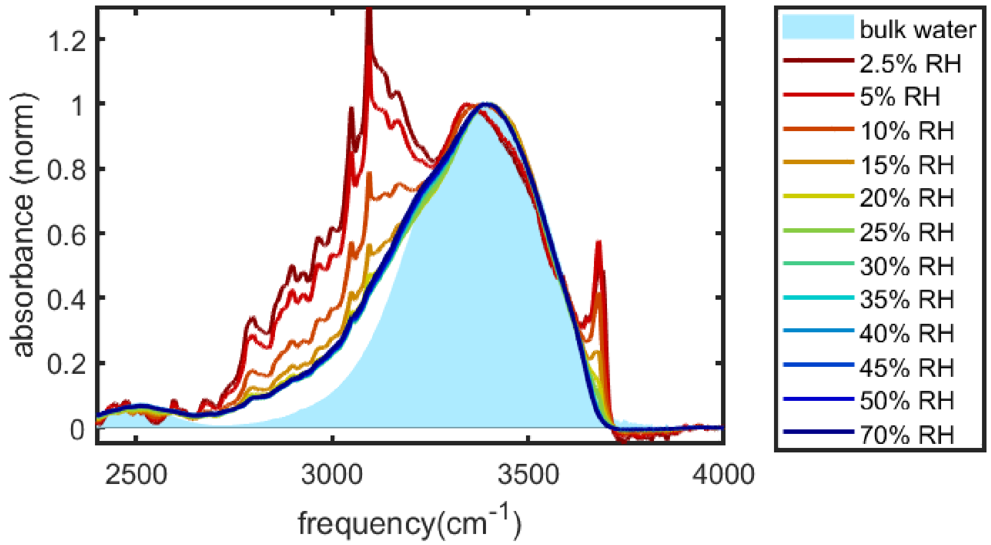

Figure S10.2: FTIR spectra of 10% HOD in Ni2Cl2BTDD at humidities from 2.5% RH to 70% RH in the O-H stretching region. The overall trends match those observed in the O-D stretching region and the O-H stretching region of H_2O, but there is interference from C-H modes and the H-O-H bending overtone obscuring the low-frequency side of the O-H stretching band.

S11: POPULATION DECAY AND HEATING EFFECT REMOVAL

Following vibrational excitation, the energy deposited into the O-D stretch by ultrafast laser pulses is converted to heat. This heat dissipates before the next laser shot, but influences the spectra enough that it must be accounted for. Here, we utilized an established fitting procedure[4] to detect and remove the heating effect from P(t) and then $C_2(t)$, taking care to avoid introducing error by overfiltering the data or using noisy data for the fit. As described in the literature,[5] the expression for P(t) is

$$P(t) = \frac{S_{\parallel}(t) + 2S_{\perp}(t)}{3}$$

(S11.1)

Where $S_{\parallel}(t)$ is the parallel signal as a function of time and $S_{\perp}(t)$ is the perpendicular signal as a function of time. P(t) is a weighted average of $S_{\parallel}(t)$ and $S_{\perp}(t)$ which results in a higher signal-to-noise ratio for P(t) than either individual measurement, so we removed the heating effect from P(t) instead of individually removing the heating effect from $S_{\parallel}(t)$ and $S_{\perp}(t)$. Removing the heating effect only once instead of twice also likely reduced the propagation of error.

To remove the heating effect from P(t), we first fit P(t) to a model with three components. The first component corresponded to the vibrationally excited state, the second component corresponded to an intermediate state, and the third component corresponded to the hot ground state observed at long *t*. After globally fitting the data, we subtracted the second and third components from the data. The fits are shown in Fig S11.1-S11.2

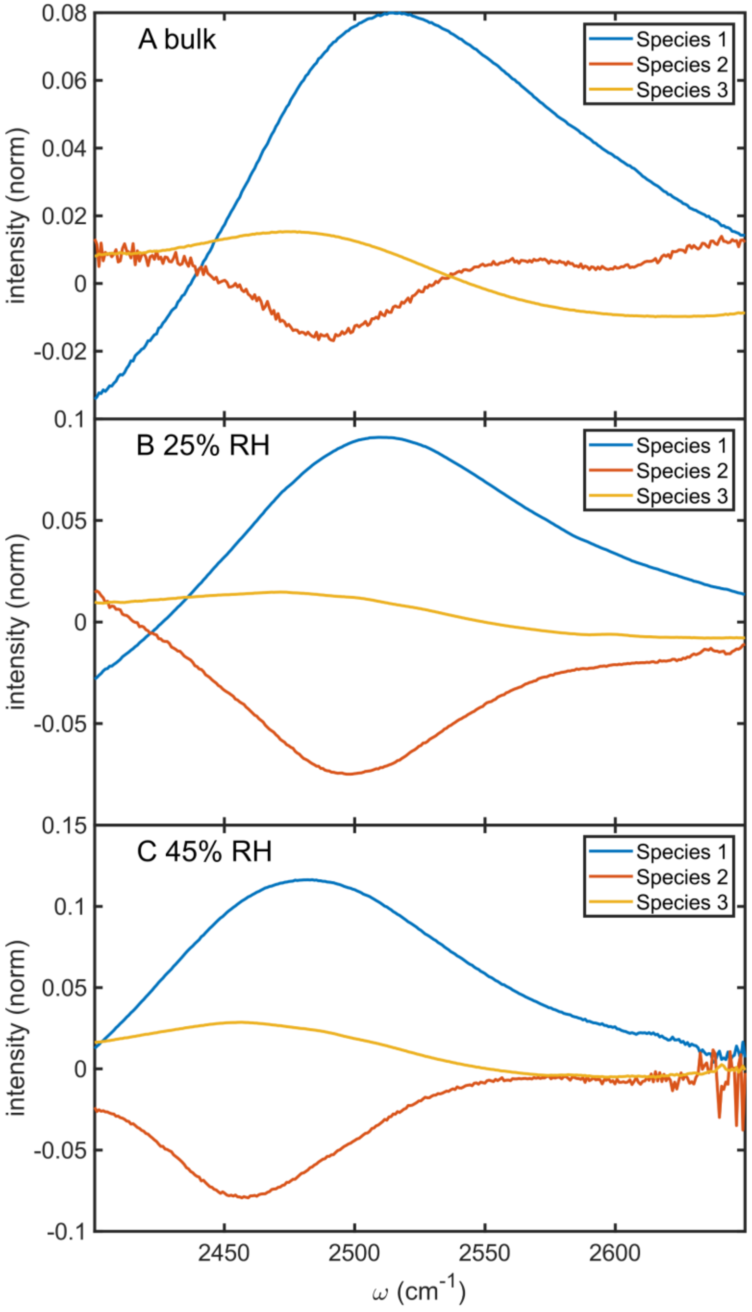

Figure S11.1. Fit components shown as a function of frequency for (A) bulk HOD, (B) HOD in Ni_2Cl_2BTDD at 25% RH, and (C) HOD in Ni_2Cl_2BTDD at 45% RH.

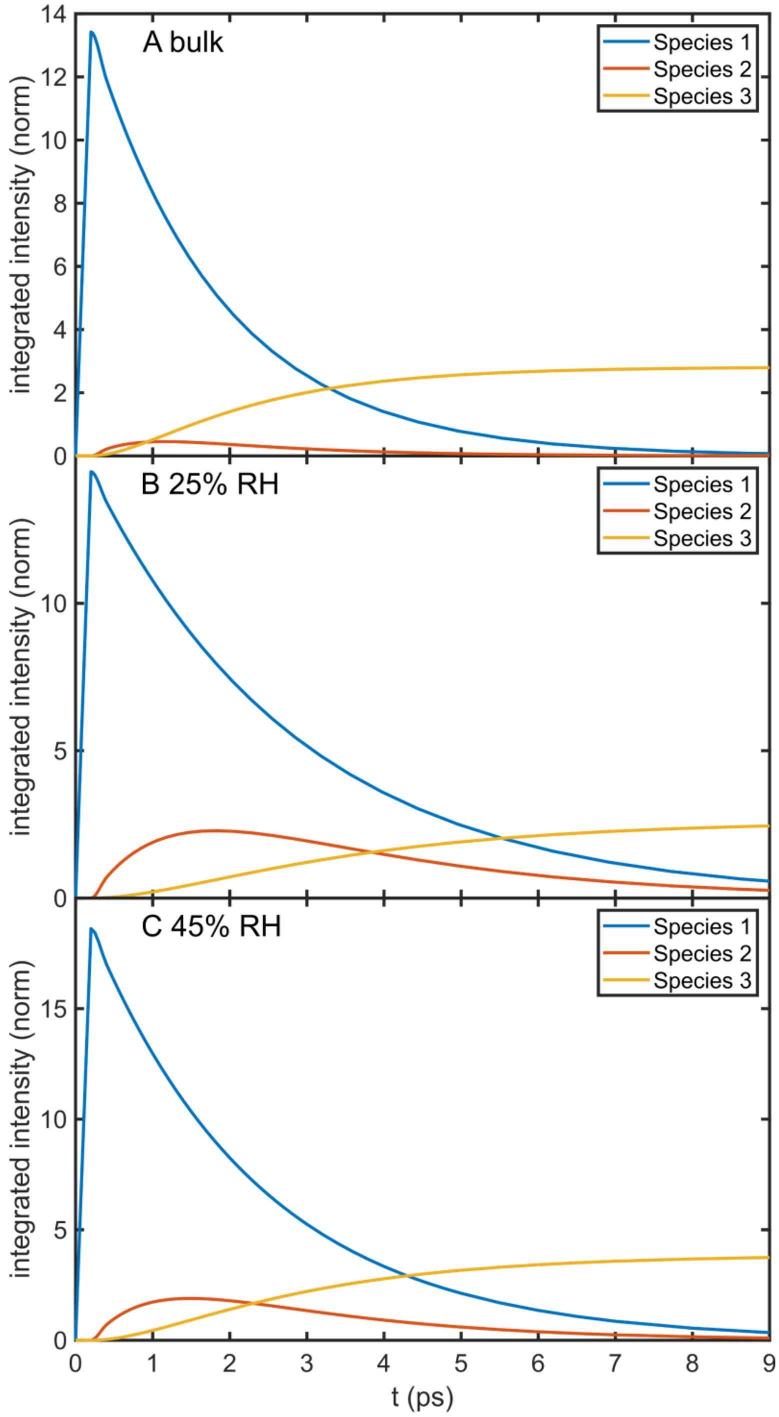

Figure S11.2. Fit components shown as a function of time delay for (A) bulk HOD, (B) HOD in Ni_2Cl_2BTDD at 25% RH, and (C) HOD in Ni_2Cl_2BTDD at 45% RH.

The population decay dynamics after correcting for the heating effect are shown in Figure S11.3 and Figure S11.4. In each system, a single vibrational lifetime was sufficient for describing the population decay dynamics.

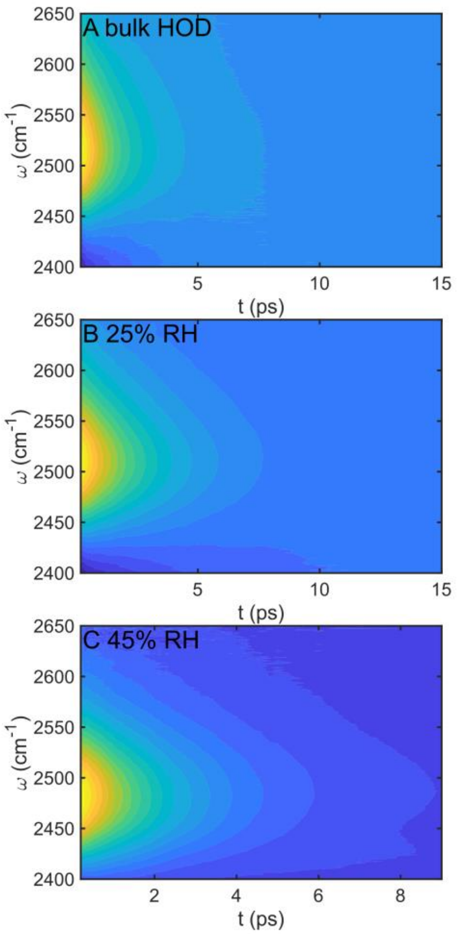

Figure S11.3 Population decay data for (A) bulk HOD, (B) HOD in Ni2Cl2BTDD at 25% RH, and (C) HOD in Ni_2Cl_2BTDD at 45% RH.

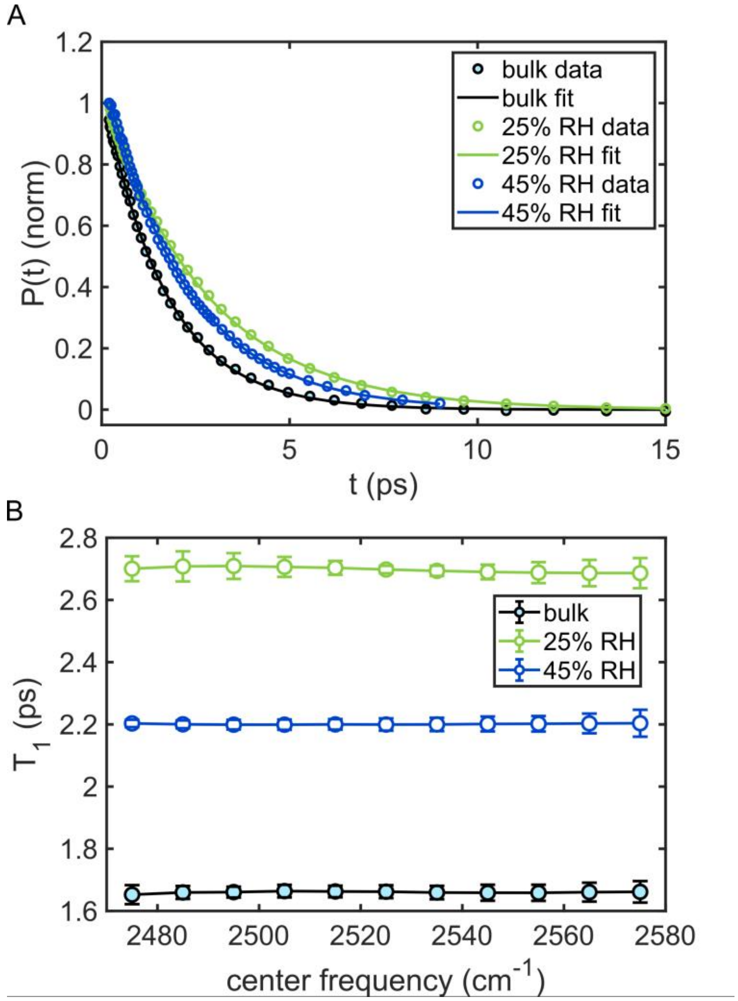

Figure S11.4: Fits to the population decay dynamics for bulk HOD (cyan), HOD in Ni_2Cl_2BTDD at 25% RH (green), and HOD in Ni2Cl2BTDD at 45% RH (blue). (A) P(t) decays at 2500-2510 cm^{-1} for all three systems with monoexponential fits. (B) Vibrational lifetimes obtained by fitting P(t) data at each frequency region.

After removing the heating effect from P(t), we were utilized the corrected P(t) data to calculate $C_2(t)$. The expression for $C_2(t)$ is

$$C_2(t) = \frac{5}{2}\left(\frac{S_{\parallel}(t) - S_{\perp}(t)}{S_{\parallel}(t) + 2S_{\perp}(t)}\right) \tag{S11.2}$$

Which can be rewritten using the expression for P(t).

$$C_2(t) = \frac{5}{6}\left(\frac{S_{\parallel}(t) - S_{\perp}(t)}{P(t)}\right)$$

(S11.3)

Here we note that the heating effect is exactly the same for $S_{\parallel}(t)$ and $S_{\perp}(t)$, so it should not impact the numerator of eq S11.2 or eq S11.3.[6] We also note that the numerator of eq S11.2 and S11.3 typically approaches small values at large values of t, which is also where the heating effect is the most significant and the signal-to-noise ratio is the lowest. As a result, we found that even small errors in estimating the heating effect from $S_{\parallel}(t)$ and $S_{\perp}(t)$ individually led to nonphysical results such as $C_2(t)$ curves that asymptotically approached negative values and failures to reproduce the literature for bulk HOD.[4,5,7] Potential errors in estimating the heating effect for P(t), however, are more minor. As discussed above, P(t) has a higher signal-to-noise ratio than $S_{\parallel}(t)$ or $S_{\perp}(t)$ and performing the heating effect removal only once instead of twice reduces the potential error. Based on the above considerations, we only removed the heating effect from P(t) in the denominator of the expression for $C_2(t)$.

S12: FÖRSTER ENERGY TRANSFER SIMULATIONS

The rotational dynamics measured using $C_2(t)$ contain contributions from both orientational motion and Förster energy transfer.[7] $C_2(t)$ is described by the following equation:

$$C_2(t) = S_0^2 \exp\left[-\frac{t}{\tau} - \frac{4\pi^{\frac{3}{2}}}{3}[OD]\sqrt{\frac{r_0^6 t}{T_1}}\right]$$

(S12.1)

Where S_0 is an order parameter that accounts for dynamics too fast to accurately measure, τ is a lifetime that describes the decay of the initial orientation, [OD] is the concentration of O-D stretches in bonds per cubic meter, r_0 is the Förster radius in meters, and T_1 is the vibrational lifetime in ps. A full description of the Förster and rotational components of $C_2(t)$ requires global fitting to $C_2(t)$ measurements made with several carefully measured values of [OD].[4,7,8] However, if approximate values for [OD], r_0, and T_1 are known, the Förster component can at least be estimated and removed from the $C_2(t)$ data as follows:

$$S_0^2 \exp\left[-\frac{t}{\tau}\right] = \frac{C_2(t)}{\exp\left[-\frac{4\pi^{\frac{3}{2}}}{3}[OD]\sqrt{\frac{r_0^6 t}{T_1}}\right]}$$

(S12.2)

Table S12.1: Parameters for simulations of Förster Energy Transfer

10% HOD system	[OD] (M)	T_1 (ps)	r_0 (Å)
Bulk water	5.56	1.7	2.3
25% RH Ni_2Cl_2BTDD	1.11	2.7	2.3
45% RH Ni_2Cl_2BTDD	3.89	2.2	2.3

Reasonable estimates can be made for [OD] in Ni_2Cl_2BTDD using the concentration of HOD molecules in a 10% solution, published isotherms of water sorption in Ni_2Cl_2BTDD,[1] and the density of liquid water. The concentration of O-D bonds in a 10% OD solution is 5.56 M, water occupies ~20% of the MOF volume at 25% RH and ~65% of the MOF volume at 45% RH.[1] Since liquid water is denser than any solid forms

of water formed near atmospheric pressure, the assumption of a 1g/mL density provides upper bounds on the concentration, and thus upper bounds on the influence of Förster energy transfer on $C_2(t)$ in MOF pores. T_1 could be approximated from P(t) data (see Section S11), and r_0 is 2.3 ± 0.2 Å for the O-D stretching

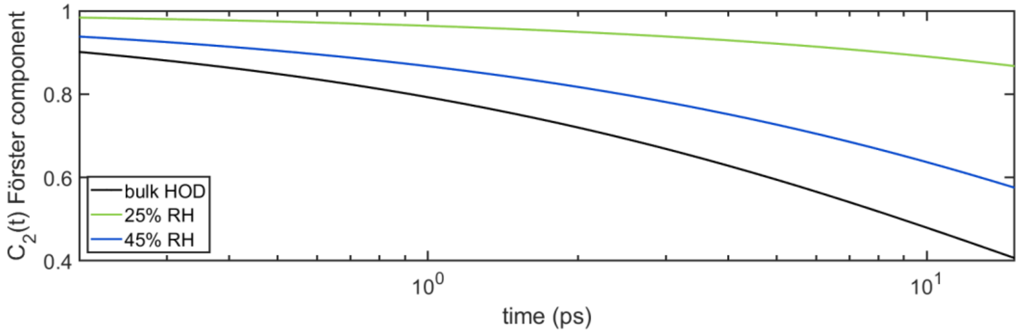

Figure S12.1: Förster Energy Transfer component of $C_2(t)$ estimated for the O-D stretch in 10% HOD in bulk water (black), 25% RH Ni_2Cl_2BTDD (green), and 45% RH Ni_2Cl_2BTDD (blue).

band.[7] Values used to model Förster energy transfer in our data are shown in Table S12.1. The estimated Förster energy transfer component of $C_2(t)$ is shown in Figure S12.1.

S13: ANISOTROPY DECAY

The anisotropy of bulk HOD could be fitted by single exponential function while the anisotropies of Ni2Cl2BTDD at 25% and 45% RH had to be fitted by single exponential function with an offset.

We used wobbling-in-the-cone model based on the reverse micelles system. So, the orientational correlation function was given by:

$$C_2(t) = (1 - T^2) \times \exp(-t/\tau_{\text{in}}) + T^2[S^2 + (1 - S^2)\exp(-t/\tau_c)] \times \exp(-t/\tau_m) \tag{S13.1}$$

The τ_{in} was too fast and τ_m was too slow to resolve. So, the correlation function could be estimated by:

$$C_2(t) = T^2[S^2 + (1 - S^2)\exp(-t/\tau_c)] \tag{S13.2}$$

The inertial cone angle θ_{in} was determined by extrapolated initial value of $C_2(t)$ at t=0. The extrapolated value was T^2 in Eq S13.2, the sum of fitted amplitude and offset. The total cone angle θ_{tot}, that was combination of inertial motion and wobbling-in-a-cone motion, was determined by T^2S^2 in Eq. S13.2, i.e. fitted offset. Then θ_{in} was obtained by Eq S13.3 with $T^2 = Q^2$. θ_{tot} was determined by Eq 13.3 with $T^2S^2 = Q^2$.

$$Q^2 = \left[\frac{1}{2}(\cos\theta)(1+\cos\theta)\right]^2$$

(S13.3)

The anisotropy fitting parameters were shown in Table S13.1. The calculated cone angles were in Table S13.2.

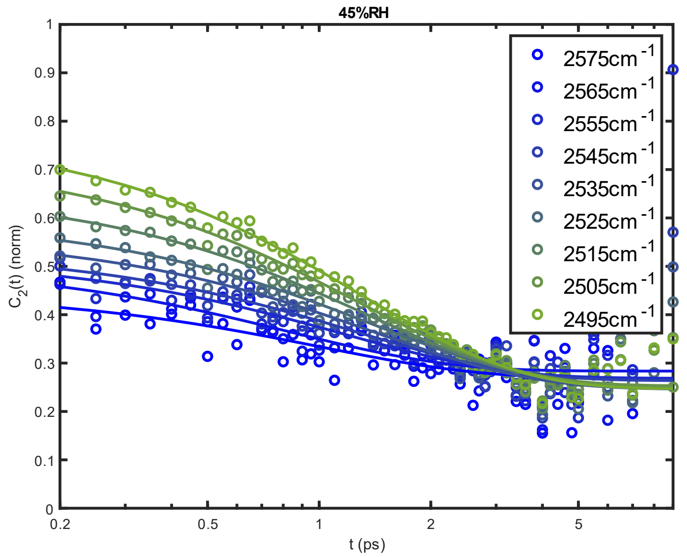

Figure S13.1 Anisotropy data for Ni_2Cl_2BTDD at 45%RH taken at each frequency region from 2495-2575 cm^{-1}.

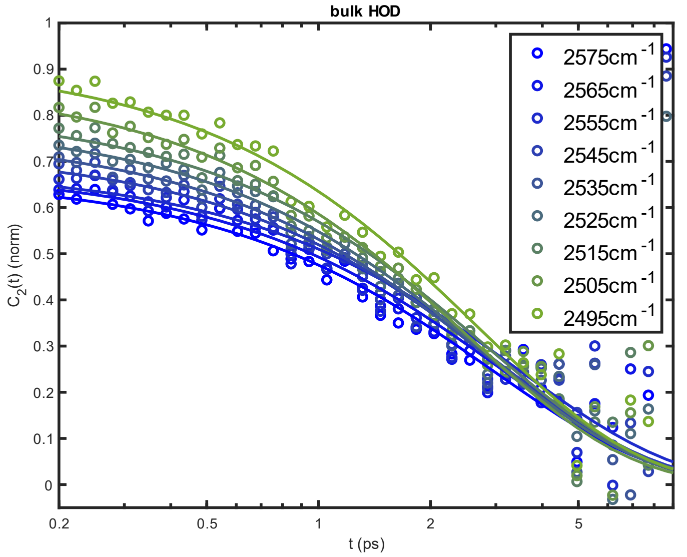

Figure S13.2 Anisotropy data for bulk 10%HOD taken at each frequency region from 2495-2575 cm^{-1}.

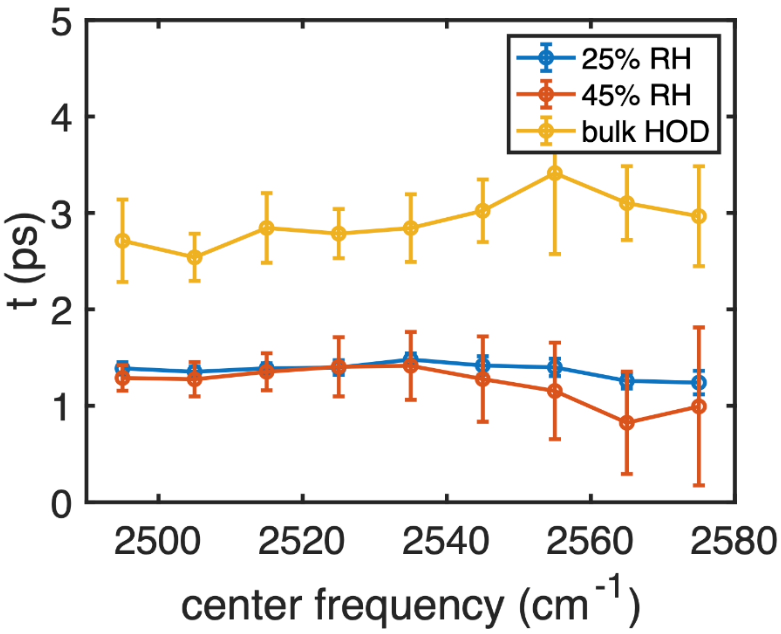

Figure S13.3 Anisotropic time constants for bulk HOD, Ni_2Cl_2BTDD at 25%RH and 45%RH. Error bars show 95% confidence intervals from fitting.

Table S13.1 Anisotropic time constants for bulk HOD, Ni_2Cl_2BTDD at 25%RH and 45%RH. Error bars show 95% confidence intervals from fitting.

10% HOD system	Amplitude	τ (ps)	offset
Bulk water	0.87±0.03	2.6±0.2	
25% RH Ni_2Cl_2BTDD	0.71±0.01	1.4±0.1	0.21±0.01
45% RH Ni_2Cl_2BTDD	0.48±0.02	1.3±0.2	0.25±0.01

Table S13.2 Cone angles calculated from anisotropy data from 2500-2510 cm^{-1} (error bars show 95% confidence intervals from fitting).

10% HOD system	θ_{in}	θ_{tot}
Bulk water	17±2	
25% RH Ni_2Cl_2BTDD	14±2	55±1

45% RH Ni_2Cl_2BTDD	26±2	52±1

S14: REFERENCES

(1) Rieth, A. J.; Wright, A. M.; Skorupskii, G.; Mancuso, J. L.; Hendon, C. H.; Dincă, M. Record-Setting Sorbents for Reversible Water Uptake by Systematic Anion Exchanges in Metal–Organic Frameworks. *J. Am. Chem. Soc.* **2019**, *141* (35), 13858–13866. https://doi.org/10.1021/jacs.9b06246.
(2) Yan, C.; Nishida, J.; Yuan, R.; Fayer, M. D. Water of Hydration Dynamics in Minerals Gypsum and Bassanite: Ultrafast 2D IR Spectroscopy of Rocks. *J. Am. Chem. Soc.* **2016**, *138* (30), 9694–9703. https://doi.org/10.1021/jacs.6b05589.
(3) Andreeva, A. B.; Le, K. N.; Kadota, K.; Horike, S.; Hendon, C. H.; Brozek, C. K. Cooperativity and Metal–Linker Dynamics in Spin Crossover Framework Fe(1,2,3-Triazolate)2. *Chem. Mater.* **2021**, *33* (21), 8534–8545. https://doi.org/10.1021/acs.chemmater.1c03143.
(4) Rezus, Y. L. A.; Bakker, H. J. On the Orientational Relaxation of HDO in Liquid Water. *J. Chem. Phys.* **2005**, *123* (11), 114502. https://doi.org/10.1063/1.2009729.
(5) Steinel, T.; Asbury, J. B.; Zheng, J.; Fayer, M. D. Watching Hydrogen Bonds Break: A Transient Absorption Study of Water. *J. Phys. Chem. A* **2004**, *108* (50), 10957–10964. https://doi.org/10.1021/jp046711r.
(6) M. Groot, C. C.; J. Bakker, H. A Femtosecond Mid-Infrared Study of the Dynamics of Water in Aqueous Sugar Solutions. *Phys. Chem. Chem. Phys.* **2015**, *17* (13), 8449–8458. https://doi.org/10.1039/C4CP05431H.
(7) Piatkowski, L.; Eisenthal, K. B.; Bakker, H. J. Ultrafast Intermolecular Energy Transfer in Heavy Water. *Phys. Chem. Chem. Phys.* **2009**, *11* (40), 9033–9038. https://doi.org/10.1039/B908975F.
(8) Woutersen, S.; Bakker, H. J. Resonant Intermolecular Transfer of Vibrational Energy in Liquid Water. *Nature* **1999**, *402* (6761), 507–509. https://doi.org/10.1038/990058.
(9) Tan, H.-S.; Piletic, I. R.; Fayer, M. D. Orientational Dynamics of Water Confined on a Nanometer Length Scale in Reverse Micelles. *J. Chem. Phys.* **2005**, *122* (17), 174501. https://doi.org/10.1063/1.1883605.
